# US Food and Drug Administration black box warnings: Characteristics of drug classes and adverse effects

**DOI:** 10.1016/j.jpet.2026.104317

**Published:** 2026-03-16

**Authors:** Lilly Josephine Bindel, Roland Seifert

**Affiliations:** Institute of Pharmacology, Hannover Medical School, Hannover, Germany

**Keywords:** Drug safety, Pharmacovigilance, US Food and Drug Administration, Black box warning, Boxed warning, Adverse effect

## Abstract

Black box warnings represent the most severe US Food and Drug Administration safety alerts. Currently, they are fragmented across individual product labels and not systematically accessible at the level of drugs or drug classes. This study provides a comprehensive overview of drugs with black box warnings and identifies class- and category-specific patterns of adverse effects, aiming to support risk benefit assessments and rational treatment. Six hundred twenty-six drugs across 250 drug classes were identified as carrying black box warnings. Drug classes were unevenly distributed, with many classes represented by a single drug, whereas a small number of large classes accounted for a substantial proportion of entries, for example, *μ*-opioid receptor agonists, cyclooxygenase inhibitors, angiotensin-converting enzyme inhibitors and kinase inhibitors. Across all drugs, 1016 adverse effect category listings were identified, demonstrating that multiple serious risks are frequently combined within a single boxed warning. Cardiac and cardiovascular adverse effects are the most prevalent category (18.7%), followed by immunologic and allergic reactions (11.4%), beside drug category-specific patterns. The standardized adverse effect count ranged from 1.1 in endocrine system drugs to 2.1 in cytotoxic treatments, indicating marked differences in the density and variety of serious adverse effects between drug categories. Black box warnings reveal pronounced heterogeneity across drugs, but show consistent, mechanism related patterns at the level of drug classes and categories. These patterns allow estimation of probable, high-risk adverse effects and risk profiles. A structured, drug class-based overview of black box warnings can improve awareness of safety risks and support rational prescribing.

**Significance Statement:**

Black box warnings are the most severe US Food and Drug Administration safety alerts, but they are fragmented across individual product labels. By systematically analyzing US Food and Drug Administration boxed warnings across drugs, drug classes, and adverse effect categories, this study reveals pronounced heterogeneity across individual drugs, but consistent, mechanism related risk patterns, with cardiovascular and immunologic adverse effects being most prevalent. A structured, drug class-based overview enables identification of high-risk profiles, supporting risk benefit assessment and rational prescribing.

## 1 Introduction

Black box warnings, or boxed warnings, “are the highest safety-related warnings that medications can have assigned by the Food and Drug Administration.”^1^ They are intended to highlight important information for prescribers, including life-threatening adverse reactions that must be considered in the risk benefit assessment before use, or risks that can be prevented by rational use and appropriate supervision, in addition to US Food and Drug Administration (FDA)-imposed restrictions of use.^2^ These warnings were introduced in 1979 and have since become an important tool for public and professional awareness.^3^ There is evidence that black box warnings influence the use of the labeled drug and related treatments.^4^^,^^5^ Given the substantial burden of medication errors,^6^ such warnings are essential to improve drug safety and treatment outcomes.^3^ However, there is evidence of high levels of nonadherence to boxed warnings by prescribers, likely because of complicated pharmacological treatments, comorbidities, and polypharmacy.^7^^,^^8^ This emphasizes the need for guidance and education.

Although the FDA provides valuable databases for drug searches, such as FDALabel,^9^ these resources mainly contain information on drug preparations and do not offer an overview of drugs or drug classes. Available literature often focuses on historical developments or on the consequences of boxed warnings for drug consumption.^3^, ^4^, ^5^ Previous research by our group has identified problematic prescribing of drugs in various conditions and countries, resulting in avoidable drug safety risks.^10^, ^11^, ^12^, ^13^

The scope of this analysis is to close this information gap by providing a comprehensive file containing information on drugs/active ingredients and their black box warnings, along with an analysis of drug and adverse effect categories to identify characteristics and high-risk profiles. This approach allows insights into drug class-specific safety profiles. By providing a systematic overview, potential adverse effects become more comprehensible for clinicians. Our aim is therefore to improve drug safety and support rational prescribing.

## 2 Materials and methods

### 2.1 Data source

Analyzed were drugs/ingredients for human use that have a black box warning label assigned by the FDA. Therefore, the official FDA database, FDALabel (https://nctr-crs.fda.gov/fdalabel/ui/search, accessed: December 20, 2025), was used. The results were filtered in the online database by “BOXED WARNINGS” (labeling section), and the full result output was downloaded as an Excel file.

### 2.2 Data extraction and clean-up

**Fig. 1:**
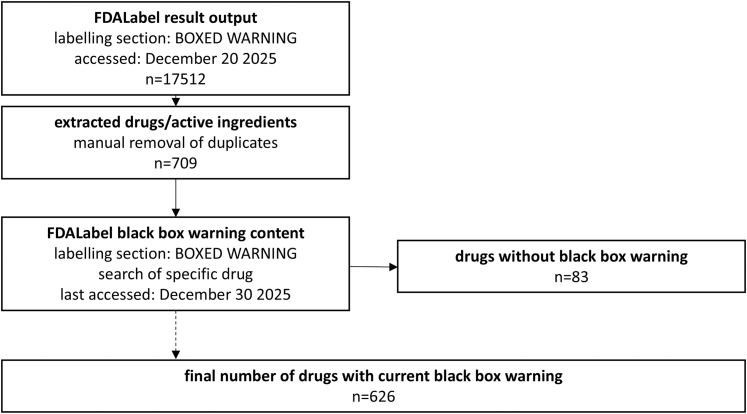
Methodological procedure for the data extraction and clean-up of drugs with black box warnings, retrieved from the FDALabel database.^7^

From the FDALabel result output, drugs were manually extracted so that each ingredient was included only once in the analysis, the results can be found in the Supplemental Table 1. Slight variations of moiety names were merged into one single listing. For example, rimabotulinumtoxin, letibotulinumtoxin, and botulinum toxin type A, were included under the single listing “botulinum toxin.” Exclusion criteria were fixed-dose combinations and excipients that do not have a pharmacological effect. A flowchart of the drug extraction process can be found in Fig. 1.

Each drug was assigned to a drug class and a drug category. The drug class was often extracted from the FDALabel output, but some entries were modified toward a more mechanistically oriented classification.^14^^,^^15^ Drug categories were created to provide a broad categorization of usage fields and to allow easier orientation and overview of the large number of listed drug classes.

Each drug was searched in the FDALabel database (https://nctr-crs.fda.gov/fdalabel/ui/search, last accessed: December 30, 2025) for black box information within the “Product Name” and the labeling section “BOXED WARNINGS.” If multiple labeling results were listed, the most recent Structured Product Labelling document was considered. If there was no listing, the drug was considered not to have a black box warning and was excluded from the drug list. Additionally, warnings were categorized into adverse effect categories and numbered consecutively to provide a searchable list of adverse effect warnings (Table 1).

**Table 1:** Overview of the categorization of black box warnings into adverse effect categories assigned to the drugs included in the analysis (see Supplemental Table 1)

No.	Adverse Effect Category	Adverse Effect Example	Drug Example
1	Hepatotoxicity and hepatic adverse effects	Liver failure, hepatomegaly with steatosis	Sunitinib
2	Nephrotoxicity and renal adverse effects	Kidney failure, nephrogenic systemic fibrosis	Gadofosveset
3	Cardiotoxicity and cardiac adverse effects	QT prolongation, cardiac arrest, hypertension	Doxorubicin
4	Neurotoxicity and neurological adverse effects	Seizures, peripheral neuropathy	Zolpidem
5	Pulmonary toxicity and respiratory adverse effects	Bronchoconstriction, respiratory depression	Terlipressin
6	Ototoxicity	Hearing loss	Gentamicin
7	Ophthalmic toxicity and visual disturbances	Vision loss	Vigabatrin
8	Gastrointestinal toxicity and gastrointestinal adverse effects	Hemorrhage, cholelithiasis	Piroxicam
9	Dermatologic toxicity and cutaneous adverse reactions	Stevens–Johnson syndrome, local tissue necrosis in extravasation	Carbamazepine
10	Musculoskeletal toxicity	Rhabdomyolysis, tendon rupture	Ciprofloxacin
11	Hematologic toxicity and hematologic adverse reactions	Myelosuppression	Chloramphenicol
12	Psychiatric and behavioral adverse effects	Suicidality	Perampanel
13	Developmental, reproductive, and embryo-fetal toxicity	Embryo-fetal toxicity, teratogenic, termination of pregnancy	Candesartan
14	Immunologic and hypersensitivity reactions	Anaphylaxis	Abacavir
15	Metabolic and endocrine adverse effects and laboratory abnormalities	Hyperkalemia, loss of bone mineral density	Desmopressin
16	Carcinogenicity and induction of malignancies	Lymphoma, skin cancer	Azathioprine
17	Increased mortality or fatal outcomes	Increased all-cause mortality	Tigecycline
18	Tolerance, dependence, misuse, and withdrawal	Addiction, abuse, misuse, dependence	Amphetamine
19	Significant drug interactions	CYP3A4	Nitroglycerin
20	Exacerbation or worsening of underlying disease	Virus reactivation	Lenacapavir
21	Infections and infection-related risks	*Clostridioides difficile* associated diarrhea, infections by meningococci	Sarilumab
22	Requirement for specialist supervision and adequate facilities	Monitoring, hospital stay	Basiliximab

For each adverse effect category, illustrative examples of drugs and adverse effects are provided. These examples are shown independently to demonstrate typical representatives within each category without an association between the listed drug and adverse effects.

### 2.3 Statistical analysis

The number and distribution of drugs, drug classes, and adverse effect categories were analyzed to observe patterns, identify distinct characteristics, and support conclusions. Besides total counts of drugs and adverse effects, a standardized adverse effect count was calculated to determine the average number of adverse effects per drug within a given drug category:

$$Standardized\;adverse\;effect\;count=\frac{total\;number\;of\;adverse\;effect\;category\;numbers}{total\;number\;of\;listed\;drugs\;per\;drug\;category}$$

The distribution of adverse effects within drug classes was calculated for each drug category and for each adverse effect category.

## 3 Results

### 3.1 Number of drugs and drug classes with black box warnings

Black box warnings are assigned to 626 drugs (allergic extract counted as one per drug class) and 250 drug classes (Table 2). For improved overview, drug classes were grouped in 9 drug categories: anti-infective drugs, cardiovascular and renal drugs, central nervous system drugs, cytotoxic treatments, diagnostic drugs, endocrine system drugs, hemostasis and blood products, immunomodulators, immunotherapies and immunization, and metabolism, gastrointestinal and respiratory system drugs. In the Supplemental Table 1, each drug is listed together with its black box warning, drug class, and assigned drug category.

**Table 2:** Number of listed drugs and drug classes per category

Drug Category	Number of Drugs	Number of Drug Classes	Number of Drugs per Drug Class		
			Average Number	Lowest Number	Largest Number
Anti-infective drugs	69	26	2.65	1	12 (NRTI)
Cardiovascular and renal drugs	76	28	2.71	1	11 (ACE inhibitor)
Central nervous system	135	42	3.21	1	19 (MOR agonist)
Cytotoxic treatment	81	28	2.89	1	12 (alkylating drug, kinase inhibitor)
Diagnostic drugs	37^a^	21	1.76	1	5 (radiographic contrast agent, paramagnetic contrast agent)
Endocrine system	35	16	2.19	1	9 (progestin)
Hemostasis and blood products	37	21	1.76	1	6 (blood coagulation factor)
Immunomodulators, immunotherapies, and immunization	104	45	2.31	1	19 (COX inhibitor)
Metabolism, gastrointestinal, and respiratory system	52	23	2.26	1	9 (hydrolytic lysosomal glucocerebroside-specific enzyme)
Sum	626	250	—	—	—
Average	69.6	27.8	2.4	1	11

Beside the absolute counts, the average, lowest, and largest number of listed drugs per drug class is presented.

ACE, angiotensin-converting enzyme; COX, cyclooxygenase; MOR, *μ*-opioid receptor; NRTI, nucleoside reverse transcriptase inhibitor.

a A methodological exception was made for allergenic extracts listed in the category “diagnostic drugs.” Here, single allergenic extracts (*n* = 201) were counted by its FDA-installed “pharmacological class” instead, in order to prevent statistical distortions by the large number of listed ingredients.

The number of included drugs and drug classes per category is lowest for the endocrine system (35 drugs and 16 drug classes) and highest for the central nervous system (135 drugs) and immunology (45 drug classes), with an average of 70 drugs and 28 drug classes per category (Table 2). The number of listed drugs per drug class ranges from 1.8 in hematology and diagnostic drugs to 3.2 for the central nervous system drugs, with an overall average of 2.4.

Although the minimum number of listed drugs per drug class is one in all categories, the maximum number varies substantially. Above the average of 11 drugs per class are drugs acting on the immune and central nervous system, with 19 representatives among cyclooxygenase inhibitors and *μ*-opioid receptor agonists. Around the average are cytotoxic treatments and anti-infective drugs, with 12 representatives of alkylating drugs, kinase inhibitors and nucleoside reverse transcriptase inhibitors, followed by cardiovascular and renal drugs, with 11 angiotensin-converting enzyme inhibitors. Below the average are the endocrine system and the metabolism, gastrointestinal and respiratory group, with 9 representatives each for progestins and hydrolytic lysosomal glucocerebroside-specific enzymes, respectively. The lowest numbers are observed for hematology, with 6 representatives of factor Xa inhibitors, and in diagnostic drugs, with 5 representatives of radiographic and paramagnetic contrast agents.

### 3.2 Adverse effect categories

Adverse effects included in boxed warnings were classified into 22 categories and numbered consecutively to facilitate searching in the Supplemental Table 1. This classification aims to highlight the biological system affected by the listed adverse effects and therefore focuses on the variety of adverse effect categories rather than the absolute number of adverse effects listed in the boxed warning.

The total number of adverse effect category listings is 1016 (Table 3), indicating that many drugs are associated with more than one adverse effect category in their boxed warnings. The highest number of listings is observed for cardiac or cardiovascular adverse events (165 drugs), followed by immunologic and allergic reactions (106 drugs).

**Table 3:** Number of adverse effect categories per drug category

No.	Adverse Effect Category	Anti-infective Drugs	Cardiovascular and Renal Drugs	Central Nervous System Drugs	Cytotoxic Treatment	Diagnostic Drugs	Endocrine System Drugs	Hemostasis and Blood Products	Immunomodulators, Immunotherapies, and Immunization	Metabolic, Gastrointestinal and Respiratory Drugs	Sum	Median
1	Hepatotoxicity or hepatic adverse effects	14	4	7	11	0	5	2	4	7	54	5
2	Nephrotoxicity or renal adverse effects	9	0	0	6	13	0	2	4	1	35	2
3	Cardiotoxicity or cardiac adverse effects	9	22	23	26	6	17	14	38	10	165	17
4	Neurotoxicity or neurological adverse effects	10	1	8	6	12	0	9	20	1	67	8
5	Pulmonary toxicity or pulmonary adverse effects	1	7	21	9	0	2	0	1	7	48	7
6	Ototoxicity	5	0	0	1	0	0	0	0	0	6	0
7	Ophthalmic toxicity	0	0	1	1	0	0	0	0	0	2	0
8	Gastrointestinal toxicity or gastrointestinal adverse effects	4	0	1	3	0	0	2	11	1	22	1
9	Dermatologic toxicity and cutaneous reactions	5	0	4	8	0	0	0	2	1	20	1
10	Musculoskeletal toxicity	4	0	1	0	5	0	0	0	1	11	1
11	Hematologic toxicity and hematologic adverse reactions	9	1	3	27	0	0	12	17	2	71	9
12	Psychiatric and behavioral effects	3	0	28	0	0	0	0	3	2	36	0
13	Developmental and reproductive toxicity	7	17	1	13	0	6	0	11	0	55	6
14	Immunologic and hypersensitivity reactions	17	3	4	12	22	1	4	24	19	106	4
15	Metabolic and endocrine effects, serum level changes	4	9	2	2	2	5	1	2	8	35	2
16	Carcinogenicity and induction of malignancies	3	1	0	9	0	2	3	17	5	40	3
17	Mortality	2	7	17	11	0	0	4	8	3	52	4
18	Tolerance, dependence, and misuse	0	0	28	0	0	0	0	0	0	28	0
19	Drug interactions	7	5	24	4	1	1	0	1	2	45	2
20	Exacerbation of disease	11	7	5	0	0	0	2	1	3	29	3
21	Infections	10	0	1	8	0	0	1	20	0	40	1
22	Supervision of professional and adequate facilities	2	5	7	13	12	0	1	7	2	49	5
	Sum	136	89	186	170	73	39	57	191	75	1016	89

Beside the absolute count, the sum and average (median) of listings is presented.

Across drug categories, the number of adverse effect category listings ranges from 39 in endocrine system drugs to 191 in immunologic drugs (Table 3). When considering the standardized adverse event count, defined as the average number of adverse effect categories per drug category, values range from 1.1 for endocrine system drugs to 2.1 for cytotoxic treatments (Table 4).

**Table 4:** Standardized adverse effect count, representing the average number of adverse effects per drug category

Drug Category	Number of Drugs	Count of Adverse Effect Categories	Standardized Adverse Effect Count
Anti-infective drugs	69	136	1.97
Cardiovascular and renal drugs	76	89	1.17
Central nervous system	135	186	1.38
Cytotoxic treatment	81	170	2.10
Diagnostic drugs	37	73	1.97
Endocrine system	35	39	1.11
Hemostasis and blood products	37	57	1.54
Immunomodulators, immunotherapies, and immunization	104	191	1.84
Metabolism, gastrointestinal, and respiratory system	52	75	1.44
Average	69.6	113	1.61

Additionally, the total number of drugs and adverse effect categories is provided.

### 3.3 Proportional distribution of adverse effects in drug categories

Beyond absolute numbers, the proportional distribution of adverse effect categories provides insight into dominant effects and allows the identification of patterns and differences between drug categories. For each drug category, the proportion of listings per adverse effect category relative to the total number of listings in that category was compared with the overall average across all drug categories.

The largest share of adverse effect listings differs between drug categories. In anti-infective drugs, the most frequently listed adverse effects are immunologic and allergic reactions (12.5%), followed by hepatotoxicity (10.3%). In cardiovascular and renal drugs, cardiac and cardiovascular effects (24.7%) and embryo-fetal toxicity (19.1%) predominate. In central nervous system drugs, psychiatric and behavioral effects, as well as tolerance, dependence, and misuse, are most prominent, accounting for 15.1% each. In cytotoxic treatments, hematologic (15.9%) and cardiovascular (15.3%) adverse effects are common. Diagnostic drugs show a high proportion of immunologic and hypersensitivity reactions (30.1%), followed by nephrotoxicity (17.8%). Endocrine system drugs have a high share of cardiac and cardiovascular effects (43.6%), followed by embryo-fetal and reproductive toxicity (15.4%). Similarly, hematologic drugs show a large share of cardiac and cardiovascular effects (24.6%), followed by hematologic toxicity (21.1%). In immunologic drugs, cardiac adverse effects (19.9%) and hypersensitivity reactions (12.6%) account for the largest proportions, whereas in drugs acting on the metabolism, gastrointestinal, and respiratory systems, hypersensitivity reactions are most frequent (25.3%), followed by cardiac adverse effects (13.3%).

**Fig. 2:**
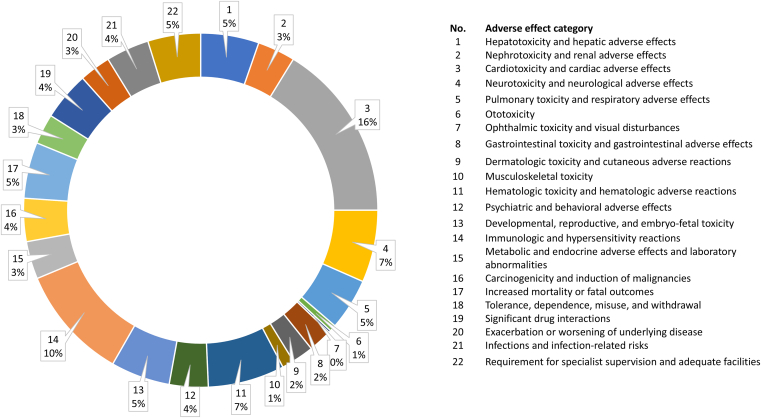
Distribution of adverse effect categories in all drugs with black box warnings (median).

Overall, the dominant adverse effect category across drug categories is cardiotoxicity/cardiovascular adverse effects (18.7% on average) (Fig. 2), being the most prevalent category in 4 drug groups (cardiovascular and renal drugs, endocrine system drugs, hematologic drugs, and immunologic drugs). This is followed by immunologic and allergic reactions (11.4% on average), which are dominant in 3 drug categories (anti-infective drugs, diagnostic drugs, and metabolism, gastrointestinal, and respiratory system drugs).

## 4 Discussion

### 4.1 Patterns of adverse effects across drug categories

Drug categories differ markedly in the number of included drugs and drug classes, with many classes represented by only 1 or 2 drugs, whereas a small number of classes account for a large proportion of entries (Table 1). The high density of a few drug classes with many representatives, such as *μ*-opioid receptor agonists, *β*-adrenergic receptor antagonists and kinase inhibitors, can be explained by their nature as large pharmacological classes with many drugs, reflecting long-standing clinical use and the development of several drug options.^16^, ^17^, ^18^, ^19^, ^20^, ^21^ Conversely, many other drug classes are represented by a single drug, reflecting innovative therapies or niche indications, such as chimeric antigen receptor T-cell therapies or antisense oligonucleotides.^22^^,^^23^ This distribution highlights a large pharmacological diversity, with established treatment options versus ongoing therapeutic innovation.^24^^,^^25^

Despite these numerical differences, drug categories show characteristic patterns with regard to adverse effects. Drugs belonging to the same drug class often share similar adverse effect profiles, as observed for norepinephrine/serotonin enhancers, *μ*-opioid receptor agonists, benzodiazepines, angiotensin-converting enzyme inhibitors, and cyclooxygenase inhibitors, among others (Table 5). Both the absolute number of adverse effect listings and the standardized adverse effect count differ across drug categories (Tables 3 and 4), allowing differentiation between the density and variety of adverse effects and facilitating the identification of high-risk drug categories.

**Table 5:** Overview of shared adverse effects in drug classes

Drug Category	Drug Class	Adverse Effect
Anti-infective drugs	Aminoglycosides	Nephrotoxicity, ototoxicity, neurotoxicity
	Penicillins, cephalosporins	Allergic reaction, *Clostridioides difficile* associated diarrhea
	Fluoroquinolones	Tendinitis and tendon rupture, myasthenia gravis exacerbation
Cardiovascular and renal drugs	ACE inhibitor	Embryo-fetal toxicity
Central nervous system drugs	Benzodiazepine	Abuse, misuse, and addiction, dependence and withdrawal reaction, problematic combination with MOR agonists
	*γ*-Aminobutyric acid A receptor agonist	Complex sleep behaviors
	NE/5-HT enhancers	Increased suicidality risk in children and young adults
	MOR agonist	Addiction, abuse, misuse, respiratory depression, neonatal opioid withdrawal syndrome, drug interactions
	mGPCR antagonist	Increased mortality in elderly patients with dementia-related psychosis
Cytotoxic treatment	Anthracycline topoisomerase inhibitor	Myelosuppression, myocardial toxicity, local tissue necrosis in extravasation
	Janus Kinase inhibitor	Infections, increased all-cause mortality; malignancies, major adverse cardiovascular events, thrombosis
Diagnostic drugs	Allergenic extracts	Anaphylaxis
Endocrine system	Estrogen	Cardiovascular risk associated with smoking
Immunomodulators, immunotherapies, and immunization	Complement inhibitor	Serious bacterial infections
	COX inhibitors	Cardiovascular and gastrointestinal adverse effects
Metabolism, gastrointestinal, and respiratory system	SGLT-2-inhibitor	Ketoacidosis
	GLP-1 Receptor Agonist	Risk of thyroid C-cell tumors

Provided are prototypical examples of drug classes and adverse effects for each drug category. More detailed information can be found in the Supplemental Table 1.

ACE, angiotensin-converting enzyme; COX, cyclooxygenase; GLP-1, glucagon-like peptide-1; mGPCR, multiple G protein-coupled receptor; MOR, *μ*-opioid receptor; NE/5-HT, norepinephrine/serotonin; SGLT2, sodium-glucose transport 2.

Cytotoxic treatments show the highest standardized adverse effect count, which is approximately twice as high as that observed for endocrine system drugs (2.1 vs 1.1) (Table 4). In general, cytotoxic drugs are associated with substantial and broad-ranging adverse effects, which complicates treatment.^26^, ^27^, ^28^ Also, systemic reactions may occur in locally applied drugs, such as anaphylaxis, in the case of allergenic extracts (Table 5; Supplemental Table 1). This illustrates that even drug categories and drugs with a relatively narrow mechanistic focus can be associated with severe adverse effect on the whole organism.

The benefit–risk ratio is a central consideration in the interpretation of black box warnings and is defined as a clinically meaningful benefit on relevant outcomes in serious or life-threatening diseases, preferably representing a clear advantage over available therapies, in combination with the ability to adequately characterize, mitigate, and manage risks.^29^^,^^30^ Severe diseases combined with proven therapeutic efficacy may justify the use of drugs despite the risk of serious adverse effects.^31^^,^^32^ However, such use requires strict monitoring and cautious application. Conversely, drugs used for diagnostic purposes or for less severe conditions, as well as drugs for which more favorable therapeutic alternatives are available, warrant more restrictive use, as seen exemplarily in the black box warnings of rizatriptan and methoxsalen or the contraindications in levothyroxine, oxytocin, and cyclooxygenase inhibitors (Supplemental Table 1).

### 4.2 Relationship between dominant adverse effects and drug mechanisms

Beside the proportional distribution of adverse effects within a drug category, it is valuable to assess whether specific adverse effects occur more or less frequently than the average across drug categories (Table 6). This comparison allows estimation of the relative prominence of certain risks and facilitates interpretation in relation to the underlying mechanisms of action.

**Table 6:** Distribution of adverse effects in drug categories

No.	Adverse Effect Category	Anti-infective Drugs, *%*	Cardiovascular and Renal Drugs, *%*	Central Nervous System Drugs, *%*	Cytotoxic Treatment, *%*	Diagnostic Drugs, *%*	Endocrine System Drugs, *%*	Hemostasis and Blood Products, *%*	Immunomodulators, Immunotherapies, and Immunization, *%*	Metabolic, Gastrointestinal, and Respiratory Drugs, *%*	Average, *%*
1	Hepatotoxicity or hepatic adverse effects	10.3	4.5	3.8	6.5	0.0	12.8	3.5	2.1	9.3	5.9
2	Nephrotoxicity or renal adverse effects	6.6	0.0	0.0	3.5	17.8	0.0	3.5	2.1	1.3	3.9
3	Cardiotoxicity or cardiac adverse effects	6.6	24.7	12.4	15.3	8.2	43.6	24.6	19.9	13.3	18.7
4	Neurotoxicity or neurological adverse effects	7.4	1.1	4.3	3.5	16.4	0.0	15.8	10.5	1.3	6.7
5	Pulmonary toxicity or pulmonary adverse effects	0.7	7.9	11.3	5.3	0.0	5.1	0.0	0.5	9.3	4.5
6	Ototoxicity	3.7	0.0	0.0	0.6	0.0	0.0	0.0	0.0	0.0	0.5
7	Ophthalmic toxicity	0.0	0.0	0.5	0.6	0.0	0.0	0.0	0.0	0.0	0.1
8	Gastrointestinal toxicity or gastrointestinal adverse effects	2.9	0.0	0.5	1.8	0.0	0.0	3.5	5.8	1.3	1.8
9	Dermatologic toxicity and cutaneous reactions	3.7	0.0	2.2	4.7	0.0	0.0	0.0	1.0	1.3	1.4
10	Musculoskeletal toxicity	2.9	0.0	0.5	0.0	6.8	0.0	0.0	0.0	1.3	1.3
11	Hematologic toxicity and hematologic adverse reactions	6.6	1.1	1.6	15.9	0.0	0.0	21.1	8.9	2.7	6.4
12	Psychiatric and behavioral effects	2.2	0.0	15.1	0.0	0.0	0.0	0.0	1.6	2.7	2.4
13	Developmental and reproductive toxicity	5.1	19.1	0.5	7.6	0.0	15.4	0.0	5.8	0.0	6.0
14	Immunologic and hypersensitivity reactions	12.5	3.4	2.2	7.1	30.1	2.6	7.0	12.6	25.3	11.4
15	Metabolic and endocrine effects, serum level changes	2.9	10.1	1.1	1.2	2.7	12.8	1.8	1.0	10.7	4.9
16	Carcinogenicity and induction of malignancies	2.2	1.1	0.0	5.3	0.0	5.1	5.3	8.9	6.7	3.8
17	Mortality	1.5	7.9	9.1	6.5	0.0	0.0	7.0	4.2	4.0	4.5
18	Tolerance, dependence, and misuse	0.0	0.0	15.1	0.0	0.0	0.0	0.0	0.0	0.0	1.7
19	Drug interactions	5.1	5.6	12.9	2.4	1.4	2.6	0.0	0.5	2.7	3.7
20	Exacerbation of disease	8.1	7.9	2.7	0.0	0.0	0.0	3.5	0.5	4.0	3.0
21	Infections	7.4	0.0	0.5	4.7	0.0	0.0	1.8	10.5	0.0	2.8
22	Supervision of professional and adequate facilities	1.5	5.6	3.8	7.6	16.4	0.0	1.8	3.7	2.7	4.8

Proportions above the average are colored blue.

Dominant and shared adverse effect regard the cardiac and immune systems, having the highest average across drug categories (Table 3; Fig. 2), with other adverse effects being more class-specific. A distinction can be made between systemic and organ-specific toxicity patterns. Systemically acting drug categories, such as cytotoxic, endocrine, and immunomodulatory drugs, affect multiple regulatory pathways^33^, ^34^, ^35^, ^36^ and therefore exhibit broader adverse effect profiles (Table 5). In contrast, targeted or exposure-limited drugs, including diagnostic and central nervous system drugs, show narrower but more pronounced effects on specific biological systems, particularly the immune system and behavior (Table 6).

The prominence of cardiac and cardiovascular adverse effects across multiple drug categories is due to the disruption of the circulatory by multiple factors.^37^ Endocrine drugs may affect cardiac function through hormonal and electrolyte dysregulation, whereas cytotoxic and immunomodulatory agents can induce myocardial injury either directly or via immune-mediated mechanisms.^38^, ^39^, ^40^ Immunologic and hypersensitivity reactions occur across several drug categories, including diagnostic and anti-infective drugs, being often driven from dysregulated immune system responses rather than from the pharmacological target.^41^^,^^42^

Distinct adverse effect patterns were observed across several drug categories. Central nervous system drugs were predominantly associated with psychiatric and behavioral effects, tolerance and drug interactions, reflecting their direct modulation of neuronal signaling and cognitive function.^43^ Cytotoxic drugs showed prominent hematologic and reproductive toxicity (Table 6), consistent with their impact on rapidly proliferating cells.^33^ Diagnostic drugs, for example, contrast media, are associated with nephrotoxicity and immune reactions, likely related to impaired renal perfusion and short-term high-dose exposure.^44^, ^45^, ^46^ However, “natural” compounds such as allergenic extracts have a similar safety profile as “synthesized” substances, with of severe adverse effects.

### 4.3 Limitations

Regarding limitations of the drug list, drug labels are continuously updated, which may result in a rapidly outdated list if it is not regularly revised. Furthermore, some drug labels are themselves in the process of being updated and may therefore still contain outdated information, as exemplified by the black box warnings for hormonal replacement therapies (Supplemental Table 1).^47^^,^^48^ In addition, boxed warnings may vary for the same drug depending on the route of administration (eg, injection vs oral) or the specific preparation (eg, fixed-dose combinations).

Several restrictions also apply to the analysis of patterns and to the categorization of drugs and adverse effect categories. The count of adverse effect items reflects the variety and density of potential adverse effects, but not their severity, as individual adverse effects are not weighted or counted separately. In addition, the frequency or prevalence of adverse events was not considered. However, detailed listings of warned adverse effects are provided in the Supplemental Table 1, allowing an approximation of severity. With respect to allocation into categories, overlaps, and alternative classifications are possible, particularly for the indication-oriented drug categories.^13^

## 5 Conclusions and future directions

The analysis of black box warnings revealed a high heterogeneity of adverse effects across drugs (Table 2, Table 3, Table 4), while showing that drugs belonging to the same mechanism-based class tend to have similar or comparable box warnings (Table 5; Supplemental Table 1). This aligns with the general principle that many adverse effects are drug class related,^49^ which is also apparent in the adverse effect profiles of broader drug categories (Table 6). This can be explained by comparable pharmacological targets and affected pathways,^50^ implying a degree of predictability and suggesting systematic rather than random effects.^51^ However, drug-specific adverse events observed in individual representatives of a drug class (Supplemental Table 1) highlight the need for individualized drug warnings and continuous pharmacovigilance.

**Fig. 3:**
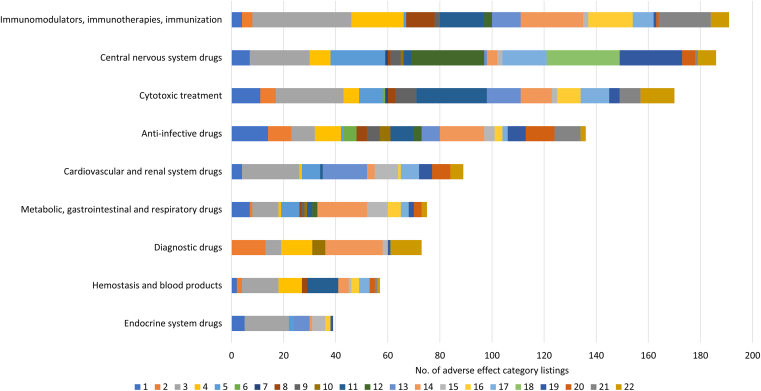
Adverse effect profiles of drug categories, providing an overview about high-risk categories and associated adverse effects. Each bar represents one drug category and is subdivided into adverse effect categories, with segment lengths corresponding to the number of listings per category. Drug categories are sorted in descending order by the total number of adverse effect category listings. Adverse effect categories are numbered as follows: (1) hepatotoxicity and hepatic adverse effects, (2) nephrotoxicity and renal adverse effects, (3) cardiotoxicity and cardiac adverse effects, (4) neurotoxicity and neurological adverse effects, (5) pulmonary toxicity and respiratory adverse effects, (6) ototoxicity, (7) ophthalmic toxicity and visual disturbances, (8) gastrointestinal toxicity and gastrointestinal adverse effects, (9) dermatologic toxicity and cutaneous adverse reactions, (10) musculoskeletal toxicity, (11) hematologic toxicity and hematologic adverse reactions, (12) psychiatric and behavioral adverse effects, (13) developmental, reproductive, and embryo-fetal toxicity, (14) immunologic and hypersensitivity reactions, (15) metabolic and endocrine adverse effects and laboratory abnormalities, (16) carcinogenicity and induction of malignancies, (17) increased mortality or fatal outcomes, (18) tolerance, dependence, misuse, and withdrawal, (19) significant drug interactions, (20) exacerbation or worsening of underlying disease, (21) infections and infection-related risks, and (22) requirement for specialist supervision and adequate facilities.

Drugs and drug classes differ in both the density and the variety of adverse effects (Tables 3 and 4), depending on their mechanism of action and the extent to which they influence biological processes. This allows an estimation of probable adverse effects, with some categories, such as cytotoxic treatments, being associated with a broad range of toxicities, whereas other drugs exhibit more organ-specific toxicities (Table 6). Nevertheless, targeted drug therapies and “natural” substances can also cause severe adverse effects, as demonstrated by targeted cytotoxic or immunologic treatments.^52^^,^^53^ An overview of high-risk drug categories and their associated adverse effect types is provided in Fig. 3. Although this knowledge does not allow prediction of future boxed warnings, it offers a structured orientation toward drug classes and categories that are predisposed to severe safety concerns, as well as toward the adverse effect types that are most likely to occur.

For rational pharmacotherapy, a benefit–risk assessment is mandatory. The treatment of severe conditions may justify the acceptance of more severe adverse events, for example in cancer therapy. Conversely, for drugs used in less severe diseases or in situations where alternative therapies are available, the occurrence of severe adverse events often leads to restricted use, as frequently observed for endocrine and anti-infective drugs (Supplemental Table 1).

Several implications for risk management can be derived from these findings. In drug labeling, it is favorable to indicate whether an adverse effect is drug class related. Furthermore, an overview of black box warnings for drugs and drug classes, created and maintained by national authorities, is needed in addition to the labeling of individual drug preparations. Although the present analysis developed such an overview, an official and publicly accessible database is required to ensure systematic and ongoing updates of drug labels. This is particularly important in the context of ongoing pharmacovigilance, as boxed warnings may change over time based on postmarketing surveillance of established drugs and newly approved treatments. Such developments are exemplarily reflected in updates to hormonal replacement therapies^47^^,^^48^ and in emerging safety signals, such as indications of carcinogenicity for glucagon-like peptide-1 receptor agonists or nitroimidazoles (Supplemental Table 1), which require further investigation and confirmation. A drug class-based approach is likely to improve pharmacovigilance as well as professional education and awareness.^54^^,^^55^

Although there are established systems for drug safety and adverse events such as Medical Dictionary for Regulatory Activities,^56^ these systems are very specialized and not suitable for broader clinical interpretation. In this context, the introduction of our 22 adverse effect categories provides a more intuitive and clinician-friendly overview of adverse events. Therefore, this study may contribute to the prevention of medication errors and support rational pharmacotherapy^57^ by providing a framework for orientation and understanding of boxed warnings. This will increase prescriber’s adherence, ultimately improving treatment outcomes and quality of life while helping to reduce the burden of medication errors.^6^

## Conflict of interest

The authors declare no conflicts of interest.
