# Supplementary material for: US Food and Drug Administration black box warnings: Characteristics of drug classes and adverse effects
Source: J Pharmacol Exp Ther. 2026 Mar 16;393(4):104317. doi: 10.1016/j.jpet.2026.104317 (PMC13197949; doi:10.1016/j.jpet.2026.104317)
Supplement: Supplementary Table 1 [file mmc1.docx]

**FDA black box warnings: characteristics of drug classes and adverse effects – Supplement**

**Lilly Josephine Bindel and Roland Seifert**

***Supplemental Table 1:*** *Comprehensive overview about all analysed drugs and their corresponding boxed warnings (last updated: December 20 2025). Provided is the drug category, class and drug name, along with the adverse reaction category and content of the black box warning. The drugs are sorted by category and class. The adverse effect categories are as follows: hepatotoxicity or hepatic adverse effects (1), nephrotoxicity or renal adverse effects (2), cardiotoxicity or cardiovascular adverse effects (3), neurotoxicity or neurological adverse effects (4), pulmonary toxicity or adverse effects (5), ototoxicity (6), ophthalmic toxicity (7), gastrointestinal toxicity or adverse effects (8), dermatologic toxicity and cutaneous reactions (9), musculoskeletal toxicity (10), hematologic toxicity and adverse reactions (11), psychiatric and behavioural effects (12), embryo-fetal and reproductive toxicity (13), immunologic and allergic reactions (14), metabolic and endocrine effects, including serum level changes (15), carcinogenicity and induction of malignancies (16), increased mortality (17), tolerance, dependence and misuse (18), drug interactions (19), exacerbation of diseases (20), infections (21), and the necessity of supervision by a professional and the availability of adequate facilities (22).*

| **Drug category** | **Drug class** | **Drug** | **Black box warning** | **Adverse reaction category** |
| --- | --- | --- | --- | --- |
| anti-infective drugs | 8-Aminoquinoline antiprotozoal | primaquine | embryo-fetal toxicity; hemolytic anemia (particularly in patients with G6PD deficiency) | 11, 13 |
| anti-infective drugs | Alkylphosphocholine antiprotozoal | miltefosine | embryo-fetal toxicity | 13 |
| anti-infective drugs | Aminoglycoside Antibacterial | streptomycin | neurotoxicity; respiratory paralysis; neuromuscular blockade | 4 |
| anti-infective drugs | Aminoglycoside Antibacterial | amikacin | ototoxicity; nephrotoxicity (aminoglycosides); neurotoxicity; neuromuscular blockade and respiratory paralysis; no combination with other neurotoxic or nephrotoxic drugs | 2, 4, 6, 19 |
| anti-infective drugs | Aminoglycoside Antibacterial | gentamicin | ototoxicity; nephrotoxicity (aminoglycosides); neurotoxicity; neuromuscular blockade and respiratory paralysis; no combination with other neurotoxic or nephrotoxic drugs; monitoring of serum concentrations | 2, 4, 6, 19 |
| anti-infective drugs | Aminoglycoside Antibacterial | neomycin | ototoxicity; nephrotoxicity (aminoglycosides); neurotoxicity; neuromuscular blockade and respiratory paralysis; no combination with other neurotoxic or nephrotoxic drugs; monitoring of serum concentrations | 2, 4, 6, 19 |
| anti-infective drugs | Aminoglycoside Antibacterial | tobramycin | ototoxicity; nephrotoxicity (aminoglycosides); neurotoxicity; neuromuscular blockade and respiratory paralysis; no combination with other neurotoxic or nephrotoxic drugs; monitoring of serum concentrations | 2, 4, 6, 19 |
| anti-infective drugs | Amphenicol-class Antibacterial | chloramphenicol | blood dyscrasias (aplastic anemia, hypoplastic anemia, thrombocytopenia, granulocytopenia) | 11 |
| ***Drug category*** | ***Drug class*** | ***Drug*** | ***Black box warning*** | ***Adverse reaction*** |
| anti-infective drugs | Azole Antifungal | itraconazole | contraindicated in heart failure; cardiac effects (negative inotropic); drug interactions (CYP3A4, CYP2D6) | 3, 19 |
| anti-infective drugs | Azole Antifungal | ketoconazole | hepatotoxicity; QT prolongation; drug interactions (CYP3A4) | 1, 3, 19 |
| anti-infective drugs | Cephalosporin Antibacterial | cefadroxil | allergic reaction (cross-hypersensitivity among beta-lactams); clostridioides difficile associated diarrhea | 14, 21 |
| anti-infective drugs | Cephalosporin Antibacterial | cefazolin | allergic reaction (cross-hypersensitivity among beta-lactams); clostridioides difficile associated diarrhea | 14, 21 |
| anti-infective drugs | Cephalosporin Antibacterial | cefoxitin | allergic reaction (cross-hypersensitivity among beta-lactams); clostridioides difficile associated diarrhea | 14, 21 |
| anti-infective drugs | Cephalosporin Antibacterial | cefuroxime | allergic reaction (cross-hypersensitivity among beta-lactams); clostridioides difficile associated diarrhea | 14, 21 |
| anti-infective drugs | Cephalosporin Antibacterial | cephalexin | allergic reaction (cross-hypersensitivity among beta-lactams); clostridioides difficile associated diarrhea | 14, 21 |
| anti-infective drugs | Cephalosporin Antibacterial | ceftazidime | allergic reaction (cross-hypersensitivity among beta-lactams); clostridioides difficile associated diarrhea; neurotoxic effects in patients with renal insufficiency (seizures, nonconvulsive status epilepticus, encephalopathy, coma, myoclonia) | 4, 14, 21 |
| anti-infective drugs | Diarylquinoline Antimycobacterial | bedaquiline | QTc prolongation | 3 |
| anti-infective drugs | Fluoroquinolone Antibacterial | gemifloxacin | tendinitis and tendon rupture | 10 |
| anti-infective drugs | Fluoroquinolone Antibacterial | ciprofloxacin | tendinitis and tendon rupture; myasthenia gravis exacerbation | 10, 20 |
| anti-infective drugs | Fluoroquinolone Antibacterial | levofloxacin | tendinitis and tendon rupture; myasthenia gravis exacerbation; peripheral neuropathy; psychiatric adverse reactions | 4, 10, 12, 20 |
| anti-infective drugs | Fluoroquinolone Antibacterial | moxifloxacin | tendinitis and tendon rupture; myasthenia gravis exacerbation; peripheral neuropathy; psychiatric adverse reactions | 4, 10, 12, 20 |
| anti-infective drugs | Glycopeptide Antibacterial | vancomycin | allergic reactions; nephrotoxicity; ototoxicity; clostridium difficile associated diarrhea | 2, 6, 14, 21 |
| anti-infective drugs | Glycopeptide Antibacterial | telavancin | increased mortalitiy in patients with renal impairment; nephrotoxicity; embryo-fetal toxicity | 2, 13, 17, |
| anti-infective drugs | Hepatitis C Virus NS5A Inhibitor | velpatasvir | hepatitis B virus reactivation in co-infections with HCV and HBV | 21 |
| anti-infective drugs | Hepatitis C Virus Nucleotide Analog NS5B Polymerase Inhibitor | sofosbuvir | hepatitis B virus reactivation in co-infections with HCV and HBV | 21 |
| anti-infective drugs | Human Immunodeficiency Virus 1 Capsid Inhibitor | nevirapine | hepatotoxicity; allergic and skin reactions (SJS, TEN, rash, organ dysfunction) | 1, 9, 14 |
| anti-infective drugs | Human Immunodeficiency Virus 1 Capsid Inhibitor | lenacapavir | risk of drug resistance for HIV-1 pre-exposure prophylaxis in undiagnosed HIV-infection | 20 |
| anti-infective drugs | Human Immunodeficiency Virus Integrase Strand Transfer Inhibitor | cabotegravir | risk of drug resistance for HIV-1 pre-exposure prophylaxis in undiagnosed HIV-infection | 20 |
| anti-infective drugs | Lincosamide Antibacterial | clindamycin | clostridioides difficile associated diarrhea | 21 |
| anti-infective drugs | Lincosamide Antibacterial | lincomycin | clostridioides difficile associated diarrhea | 21 |
| ***Drug category*** | ***Drug class*** | ***Drug*** | ***Black box warning*** | ***Adverse reaction*** |
| anti-infective drugs | Macrolide antibacterial | azithromycin | allergic and skin reaction; hepatotoxicity; infantile hypertrophic pyloric stenosis in children; QT prolongation and torsades de pointes; cardiovascular death; clostridioides difficile associated diarrhea; exacerbation of myasthenia gravis | 1, 3, 8, 14, 20, 21 |
| anti-infective drugs | Macrolide antibacterial | telithromycin | contraindicated in myasthenia gravis (respiratory failure) | 5, 20 |
| anti-infective drugs | Macrolide antibacterial | clarithromycin | severe acute hypersensitivity reaction; QT prolongation; hepatotoxicity; clostridium difficile associated diarrhea; embryo-fetal toxicity; exacerbation of myasthenia gravis | 1, 3, 13, 14, 20, 21 |
| anti-infective drugs | Mycolic acid synthesis inhibitor | isoniazid | severe hepatitis | 1, 14 |
| anti-infective drugs | Nitroimidazole | metronidazole | carcinogenic (proved in animals, uncertain for humans) | 16 |
| anti-infective drugs | Nitroimidazole | tinidazole | carcinogenic (proved in metronidazole in animals, uncertain for humans and tinidazole but similar characteristics of drugs) | 16 |
| anti-infective drugs | Nucleoside Analog Antifungal | flucytosine | extreme caution in patients with impaired renal function and bone marrow depression (toxicity) | 20 |
| anti-infective drugs | Nucleoside Analog Antiviral | ribavirin | embryo-fetal toxicity; hemolytic anemia and worsening of cardiac disease (myocardial infarctions); no monotherapy (ineffective and contraindicated) | 3, 11, 13, 20 |
| anti-infective drugs | Nucleoside Reverse Transcriptase Inhibitor | abacavir | hypersensitivity reactions (high risk and contraindicated in HLA-B*5701 patients) | 14 |
| anti-infective drugs | Nucleoside Reverse Transcriptase Inhibitor | dolutegravir | hypersensitivity reactions (rash, constitutional findings, organ dysfunction, liver injury); hepatotoxicity; embryo-fetal toxicity; immune reconstitution syndrome | 1, 13, 14 |
| anti-infective drugs | Nucleoside Reverse Transcriptase Inhibitor | adefovir | nephrotoxicity; HIV resistance; lactic acidosis and hepatomegaly with steatosis; post treatment acute exacerbation of hepatitis B | 1, 2, 15, 20, 21 |
| anti-infective drugs | Nucleoside Reverse Transcriptase Inhibitor | didanosine | pancreatitis; lactic acidosis; hepatomegaly with steatosis | 1, 8, 15 |
| anti-infective drugs | Nucleoside Reverse Transcriptase Inhibitor | stavudine | pancreatitis; lactic acidosis; hepatomegaly with steatosis | 1, 8, 15 |
| anti-infective drugs | Nucleoside Reverse Transcriptase Inhibitor | bictegravir | post treatment acute exacerbation of hepatitis B | 20 |
| anti-infective drugs | Nucleoside Reverse Transcriptase Inhibitor | rilpivirine | post treatment acute exacerbation of hepatitis B | 20 |
| anti-infective drugs | Nucleoside Reverse Transcriptase Inhibitor | tenofovir | post treatment acute exacerbation of hepatitis B | 20 |
| anti-infective drugs | Nucleoside Reverse Transcriptase Inhibitor | zidovudine | post treatment acute exacerbation of hepatitis B; lactic acidosis and hepatomegaly with steatosis; hematologic toxicity (neutropenia, anemia) | 1, 11, 15, 20 |
| anti-infective drugs | Nucleoside Reverse Transcriptase Inhibitor | lamivudine | post treatment acute exacerbation of hepatitis B; lactic acidosis and hepatomegaly; hematologic toxicity (neutropenia, anemia) | 1, 11, 15, 20 |
| anti-infective drugs | Nucleoside Reverse Transcriptase Inhibitor | entecavir | post treatment acute exacerbation of hepatitis B; lactic acidosis and hepatomegaly; risk of drug resistance in co-infection with HIV and HBV | 20, 21 |
| anti-infective drugs | Nucleoside Reverse Transcriptase Inhibitor | emtricitabine | post treatment acute exacerbation of hepatitis B; risk of drug resistance for HIV-1 pre-exposure prophylaxis in undiagnosed HIV-infection | 20, 21 |
| anti-infective drugs | Penicillin-class Antibacterial | piperacillin | allergic reaction (cross-hypersensitivity among beta-lactams) | 14 |
| anti-infective drugs | Penicillin-class Antibacterial | tazobactam | allergic reaction (cross-hypersensitivity among beta-lactams) and severe cutaneous adverse reactions (TEN, SJS, exanthema); hemophagocytic lymphohistiocytosis; rhandomyolysis; neuromuscular excitability and seizures; nephrotoxicity; clostridioides difficile associated diarrhea | 2, 4, 9, 10, 14, 21 |
| ***Drug category*** | ***Drug class*** | ***Drug*** | ***Black box warning*** | ***Adverse reaction*** |
| anti-infective drugs | Penicillin-class Antibacterial | sulbactam | allergic reaction (cross-hypersensitivity among beta-lactams) and severe cutaneous adverse reactions (TEN,m SJS, AGEP); hepatotoxicity (hepatitis, cholestatic jaundice); clostridium difficile associated diarrhea | 1, 9, 14, 21 |
| anti-infective drugs | Penicillin-class Antibacterial | ampicillin | allergic reaction (cross-hypersensitivity among beta-lactams); clostridioides difficile associated diarrhea | 14, 20 |
| anti-infective drugs | Penicillin-class Antibacterial | penicillin G | allergic reaction (cross-hypersensitivity among beta-lactams); clostridioides difficile associated diarrhea | 14, 20 |
| anti-infective drugs | Penicillin-class Antibacterial | amoxicillin | allergic reaction (cross-hypersensitivity among beta-lactams); clostridioides difficile associated diarrhea | 14, 20 |
| anti-infective drugs | Polyene Antifungal | amphotericin B | cardiac or cardiopulmonary arrest in overdosage | 3 |
| anti-infective drugs | Polymyxin-class Antibacterial | polymyxin B | supervision by a trained professional; nephrotoxicity; dosage adjustment in patients with renal damage; neurotoxicity | 2, 4, 22 |
| anti-infective drugs | Protease Inhibitor | ritonavir | drug interactions (CYP3A4) | 19 |
| anti-infective drugs | Protease Inhibitor | nirmatrelvir | drug interactions with paxlovid and CYP3A4 | 19 |
| anti-infective drugs | Protease Inhibitor | darunavir | hepatitis; skin reaction (SJHS, TEN, rash with eosinophilia, exanthema); allergic reactions (cross-hypersensitivity with sulfonamides); diabetes mellitus or hyperglycemia; redistribution/accumulation of body fat; immune reconstitution syndrome; bleeding events in patients with hemophilia; contraindicated in children under 3 years because toxicity | 1, 9, 11, 14, 15 |
| anti-infective drugs | Protease Inhibitor | tipranavir | hepatotoxicitiy; intracranial hemorrhage | 1, 11 |
| anti-infective drugs | Protease Inhibitor | elvitegravir | post treatment acute exacerbation of hepatitis B (in co-infected patients with HIV and HBV) | 20 |
| anti-infective drugs | Protease Inhibitor | atazanavir | PR interval prolongation; skin reactions; hyperbilirubinemia; hepatotoxicity; nephrotoxicity; nephrolithiasis and cholelithiasis; diabetes mellitus and hyperglycemia; immune reconstitution syndrome; redistribution/accumulation of body fat; drug interactions | 1, 2, 3, 8, 9, 14, 15, 19 |
| anti-infective drugs | Quinoline heme-polymerization inhibitor | quinine | hematologic reactions (thrombocytopenia, HUS/TTP) | 11, 14 |
| anti-infective drugs | Quinoline heme-polymerization inhibitor | mefloquine | neuropsychiatric adverse reactions | 12 |
| anti-infective drugs | Tetracycline-class Antibacterial | tigecycline | increased all-cause mortality | 17 |
| anti-infective drugs | Viral DNA Polymerase Inhibitor | valganciclovir | hematologic toxicity (leukopenia, neutropenia, anemia, thrombocytopenia, pancytopenia, aplastic anemia); impairment of fertility; embryo-fetal toxicity; carcinogenic | 2, 11, 13, 16 |
| anti-infective drugs | Viral DNA Polymerase Inhibitor | cidofovir | renal toxicity; neutropenia; carcinogenic; teratogenic; hypospermia | 2, 11, 13, 16 |
| cardiovascular and renal drugs | Aldosterone Antagonist | spironolactone | carcinogenic (proved in animals, uncertain for humans) | 16 |
| cardiovascular and renal drugs | Aldosterone Antagonist | eplerenone | hyperkalemia | 15 |
| cardiovascular and renal drugs | alpha- and beta-Adrenergic Agonist | droxidopa | supine hypertension | 3 |
| cardiovascular and renal drugs | alpha-Adrenergic Agonist | midodrine | supine hypertension | 3 |
| ***Drug category*** | ***Drug class*** | ***Drug*** | ***Black box warning*** | ***Adverse reaction*** |
| cardiovascular and renal drugs | Angiotensin 2 Receptor Antagonist | azilsartan | embryo-fetal toxicity | 13 |
| cardiovascular and renal drugs | Angiotensin 2 Receptor Antagonist | candesartan | embryo-fetal toxicity | 13 |
| cardiovascular and renal drugs | Angiotensin 2 Receptor Antagonist | eprosartan | embryo-fetal toxicity | 13 |
| cardiovascular and renal drugs | Angiotensin 2 Receptor Antagonist | irbesartan | embryo-fetal toxicity | 13 |
| cardiovascular and renal drugs | Angiotensin 2 Receptor Antagonist | losartan | embryo-fetal toxicity | 13 |
| cardiovascular and renal drugs | Angiotensin 2 Receptor Antagonist | olmesartan | embryo-fetal toxicity | 13 |
| cardiovascular and renal drugs | Angiotensin 2 Receptor Antagonist | valsartan | embryo-fetal toxicity | 13 |
| cardiovascular and renal drugs | Angiotensin 2 Receptor Antagonist | amlodipine | potentially embryo-fetal toxic | 13 |
| cardiovascular and renal drugs | Angiotensin Converting Enzyme Inhibitor | benazepril | embryo-fetal toxicity | 13 |
| cardiovascular and renal drugs | Angiotensin Converting Enzyme Inhibitor | captopril | embryo-fetal toxicity | 13 |
| cardiovascular and renal drugs | Angiotensin Converting Enzyme Inhibitor | enalapril | embryo-fetal toxicity | 13 |
| cardiovascular and renal drugs | Angiotensin Converting Enzyme Inhibitor | fosinopril | embryo-fetal toxicity | 13 |
| cardiovascular and renal drugs | Angiotensin Converting Enzyme Inhibitor | lisinopril | embryo-fetal toxicity | 13 |
| cardiovascular and renal drugs | Angiotensin Converting Enzyme Inhibitor | moexipril | embryo-fetal toxicity | 13 |
| cardiovascular and renal drugs | Angiotensin Converting Enzyme Inhibitor | perindopril | embryo-fetal toxicity | 13 |
| cardiovascular and renal drugs | Angiotensin Converting Enzyme Inhibitor | quinapril | embryo-fetal toxicity | 13 |
| cardiovascular and renal drugs | Angiotensin Converting Enzyme Inhibitor | ramipril | embryo-fetal toxicity | 13 |
| cardiovascular and renal drugs | Angiotensin Converting Enzyme Inhibitor | trandolapril | embryo-fetal toxicity | 13 |
| cardiovascular and renal drugs | Angiotensin Converting Enzyme Inhibitor | telmisartan | embryo-fetal toxicity | 13 |
| cardiovascular and renal drugs | beta2-Adrenergic Agonist | terbutaline | contraindicated for tocolysis | 17 |
| cardiovascular and renal drugs | beta2-Adrenergic Agonist | formoterol | increased risk of asthma related death | 5, 17 |
| cardiovascular and renal drugs | beta2-Adrenergic Agonist | salmeterol | increased risk of asthma related death | 5, 17 |
| cardiovascular and renal drugs | beta2-Adrenergic Agonist | albuterol | paradoxical bronchospasm; cardiovascular effects; hypersensitivity reactions; hypokalemia; changes in blood glucose | 3, 5, 14, 15 |
| ***Drug category*** | ***Drug class*** | ***Drug*** | ***Black box warning*** | ***Adverse reaction*** |
| cardiovascular and renal drugs | beta2-Adrenergic Agonist | levalbuterol | paradoxical bronchospasm; cardiovascular effects; hypersensitivity reactions; hypokalemia; changes in blood glucose | 3, 5, 14, 15 |
| cardiovascular and renal drugs | beta-Adrenergic Antagonist | sotalol | availability of trained professionals and adequate facilities | 20 |
| cardiovascular and renal drugs | beta-Adrenergic Antagonist | atenolol | no abrupt discontinuation (resulting in exacerbation of angina pectoris; myocardial infarction; ventricular arrhythmias) | 20 |
| cardiovascular and renal drugs | beta-Adrenergic Antagonist | metoprolol | no abrupt discontinuation (resulting in exacerbation of angina pectoris; myocardial infarction; ventricular arrhythmias) | 20 |
| cardiovascular and renal drugs | beta-Adrenergic Antagonist | nadolol | no abrupt discontinuation (resulting in exacerbation of angina pectoris; myocardial infarction; ventricular arrhythmias) | 20 |
| cardiovascular and renal drugs | beta-Adrenergic Antagonist | propranolol | no abrupt discontinuation (resulting in exacerbation of angina pectoris; myocardial infarction; ventricular arrhythmias) | 20 |
| cardiovascular and renal drugs | Cardiac Myosin Inhibitor | mavacamten | risk of heart failure (systolic dysfunction) | 3 |
| cardiovascular and renal drugs | Dihydropyridine Calcium Channel Blocker | nimodipine | drug interactions (CYP3A4); contraindicated in parenteral routes | 19 |
| cardiovascular and renal drugs | Dihydropyridine Calcium Channel Blocker | nifedipine | excessive hypotension; increased angina and myocardial infarction; congestive heart failure | 3 |
| cardiovascular and renal drugs | Endothelin Receptor Antagonist | ambrisentan | embryo-fetal toxicity | 13 |
| cardiovascular and renal drugs | Endothelin Receptor Antagonist | aprocitentan | embryo-fetal toxicity | 13 |
| cardiovascular and renal drugs | Endothelin Receptor Antagonist | atrasentan | embryo-fetal toxicity | 13 |
| cardiovascular and renal drugs | Endothelin Receptor Antagonist | macitentan | embryo-fetal toxicity | 13 |
| cardiovascular and renal drugs | Endothelin Receptor Antagonist | bosentan | embryo-fetal toxicity; hepatotoxicity | 1, 13 |
| cardiovascular and renal drugs | Endothelin Receptor Antagonist | sparsentan | embryo-fetal toxicity; hepatotoxicity | 1, 13 |
| cardiovascular and renal drugs | Ergotamine Derivative | dihydroergotamine | peripheral ischemia following co-administration with potent CYP3A4 inhibitors | 19 |
| cardiovascular and renal drugs | NCC inhibitor | hydrochlorothiazide | embryo-fetal toxicity | 13 |
| cardiovascular and renal drugs | NCC inhibitor | metolazone | rapid onset hyponatremia and hypokalemia; drug interactions (particularly lithium, furosemide, other antihypertensive drugs); cross-allergy (sulfonamides, thiazides, quinethazones); sensitivity reactions; no interchanging of drug preparation | 14, 15, 19 |
| cardiovascular and renal drugs | Neprilysin Inhibitor | sacubitril | embryo-fetal toxicity | 13 |
| cardiovascular and renal drugs | NKCC inhibitor | bumetanide | diuresis and electrolyte depletion; supervision of trained professional | 15 |
| cardiovascular and renal drugs | NKCC inhibitor | furosemide | diuresis and electrolyte depletion; supervision of trained professional | 15 |
| cardiovascular and renal drugs | NKCC inhibitor | triamterene | elevation of serum potassium levels; hyperkalemia | 15 |
| ***Drug category*** | ***Drug class*** | ***Drug*** | ***Black box warning*** | ***Adverse reaction*** |
| cardiovascular and renal drugs | NO Donor | nitroglycerin | drug interactions (amplification of vasodilatory effects by phosphodiesterase inhibitors) | 19 |
| cardiovascular and renal drugs | NO Donor | nitrite ion | hypotension; methemoglobin formation; supervision of trained professional and availability of adequate facilities | 3, 15 |
| cardiovascular and renal drugs | NO Donor | nitroprusside | hypotension; toxicity (cyanide ion); supervision by a trained professional and availability of adequate facilities | 3, 22 |
| cardiovascular and renal drugs | Osmotic Diuretic | mannitol | risk of severe bronchospasm; supervision of trained professional and availability of adequate facilities | 5, 22 |
| cardiovascular and renal drugs | Phosphodiesterase 3 Inhibitor | sildenafil | cardiac risk in sexual activity with cadiovascular disease | 3, 20 |
| cardiovascular and renal drugs | Phosphodiesterase 3 Inhibitor | cilostazol | contraindicated in heart failure | 20 |
| cardiovascular and renal drugs | Phosphodiesterase 5 Inhibitor | tadalafil | cardiovascular effects; drug interactions (amplification of vasodilatory effects in combination with nitrates) | 3, 19 |
| cardiovascular and renal drugs | Pleiotropic Channel Blocker | dronedarone | increased risk of death, stroke and heart failure in patients with permanent atrial fibrillation; contraindication for advanced heart failure and permanent atrial fibrillation | 3, 17, 20 |
| cardiovascular and renal drugs | Pleiotropic Channel Blocker | amiodarone | pulmonary toxicity; hepatotoxicity; cardiac toxicity (exacerbation of arrhythmias) | 1, 3, 5, 20 |
| cardiovascular and renal drugs | Potassium Channel Blocker | ibutilide | arrhythmias (torsades de pointes, QT prolongation); availability of trained professional and adequate facilities | 3, 22 |
| cardiovascular and renal drugs | Potassium Channel Blocker | dofetilide | ventricular arrhythmia (torsade des pointes); availability of trained professionals and adequate facilities | 3, 22 |
| cardiovascular and renal drugs | Potassium Channel Opener | minoxidil | adverse cardiac effects: pericardial effusion; tamponade; exacerbation of angina pectoris; myocardial lesions; availability of trained professionals and adequate facilities | 3, 20, 22 |
| cardiovascular and renal drugs | Prostaglandin Analog | alprostadil | apnea (neonates); supervision of trained professional and availability of adequate facilities | 5, 22 |
| cardiovascular and renal drugs | Prostaglandin Analog | carboprost | supervision of trained professional and availability of adequate facilities; strict adherence to recommended dosing | 20 |
| cardiovascular and renal drugs | Protease-activated Receptor-1 Antagonist | vorapaxar | bleeding risk | 11 |
| cardiovascular and renal drugs | Renin Inhibitor | aliskiren | embryo-fetal toxicity | 13 |
| cardiovascular and renal drugs | Sodium Channel Blocker | propafenone | increased mortalitiy and nonfatal cardiac arrest rate | 3, 17 |
| cardiovascular and renal drugs | Sodium Channel Blocker | mexiletine | increased mortalitiy and nonfatal cardiac arrest rate; acute liver injury | 1, 3, 17 |
| cardiovascular and renal drugs | Sodium Channel Blocker | disopyramide | increased mortalitiy and nonfatal cardiac arrest rate; proarrhythmic; negative inotropic (heart failure, hypotension); QRS widening and QT prolongation; hypoglycemia; anticholinergic activity | 3, 4, 15, 17 |
| cardiovascular and renal drugs | Sodium Channel Blocker | flecainide | increased mortalitiy and nonfatal cardiac arrest rate; ventricular arryhtmias in patients with atrial fibrillation/flutter | 3, 17, 20 |
| cardiovascular and renal drugs | Sodium Channel Blocker | quinidine | increased mortality rates | 17 |
| cardiovascular and renal drugs | Sodium Channel Blocker | procainamide | positive ANA test with or without symptoms of a lupus erythematosus-like syndrome | 14 |
| ***Drug category*** | ***Drug class*** | ***Drug*** | ***Black box warning*** | ***Adverse reaction*** |
| cardiovascular and renal drugs | Sodium-Hydrogen Exchanger 3 Inhibitor | riociguat | embryo-fetal toxicity | 13 |
| cardiovascular and renal drugs | Soluble Guanylate Cyclase Stimulator | vericiguat | embryo-fetal toxicity | 13 |
| cardiovascular and renal drugs | Vasopressin Receptor Agonist | desmopressin | hyponatremia | 15 |
| cardiovascular and renal drugs | Vasopressin Receptor Agonist | terlipressin | respiratory failure | 5 |
| cardiovascular and renal drugs | Vasopressin V2 Receptor Antagonist | tolvaptan | contraindicated in autosomal dominant polycystic kidney disease (hepatotoxicity); osmotic demyelination in too rapid correction of hyponatremia; supervision of trained professional and availability of adequate facilities | 1, 4 |
| central nervous system | Acetylcholine Release Inhibitor | botulinum toxin | distant spread of toxin effect | 9 |
| central nervous system | AChR Agonist | varenicline | serious neuropsychiatric events (agitation, hostility, depression, changes in behaviour or thinking, suicidality) | 12 |
| central nervous system | AChR Agonist | methacholine | severe bronchoconstriction (contraindicated in asthma, wheezing or FEV1 below 60%) | 5 |
| central nervous system | AChR Agonist | histamine | use only under supervision of a physician | 22 |
| central nervous system | Alkali Ion | lithium | toxicity and narrow therapeutic range; facility for monitoring | 22 |
| central nervous system | Aminoketone | bupropion | increased suicidality risk in children and young adults | 12 |
| central nervous system | Barbiturate | phenobarbital | abuse, misuse and addiction; dependence and withdrawal reaction; problematic combination with MOR agonists | 18, 19 |
| central nervous system | Barbiturate | methohexital | personnel and equipment for monitoring and resuscitation | 3, 22 |
| central nervous system | Benzodiazepine | alprazolam | abuse, misuse and addiction; dependence and withdrawal reaction; problematic combination with MOR agonists | 18, 19 |
| central nervous system | Benzodiazepine | chlordiazepoxide | abuse, misuse and addiction; dependence and withdrawal reaction; problematic combination with MOR agonists | 18, 19 |
| central nervous system | Benzodiazepine | clobazam | abuse, misuse and addiction; dependence and withdrawal reaction; problematic combination with MOR agonists | 18, 19 |
| central nervous system | Benzodiazepine | clonazepam | abuse, misuse and addiction; dependence and withdrawal reaction; problematic combination with MOR agonists | 18, 19 |
| central nervous system | Benzodiazepine | clorazepate dipotassium | abuse, misuse and addiction; dependence and withdrawal reaction; problematic combination with MOR agonists | 18, 19 |
| central nervous system | Benzodiazepine | diazepam | abuse, misuse and addiction; dependence and withdrawal reaction; problematic combination with MOR agonists | 18, 19 |
| central nervous system | Benzodiazepine | estazolam | abuse, misuse and addiction; dependence and withdrawal reaction; problematic combination with MOR agonists | 18, 19 |
| central nervous system | Benzodiazepine | lorazepam | abuse, misuse and addiction; dependence and withdrawal reaction; problematic combination with MOR agonists | 18, 19 |
| central nervous system | Benzodiazepine | oxazepam | abuse, misuse and addiction; dependence and withdrawal reaction; problematic combination with MOR agonists | 18, 19 |
| central nervous system | Benzodiazepine | temazepam | abuse, misuse and addiction; dependence and withdrawal reaction; problematic combination with MOR agonists | 18, 19 |
| ***Drug category*** | ***Drug class*** | ***Drug*** | ***Black box warning*** | ***Adverse reaction*** |
| central nervous system | Benzodiazepine | triazolam | abuse, misuse and addiction; dependence and withdrawal reaction; problematic combination with MOR agonists | 18, 19 |
| central nervous system | Benzodiazepine | midazolam | personnel and equipment for monitoring and resuscitation; abuse, misuse and addiction; dependence and withdrawal reaction; problematic combination with MOR agonists | 3, 18, 19, 22 |
| central nervous system | Benzodiazepine | remimazolam | personnel and equipment for monitoring and resuscitation; abuse, misuse and addiction; dependence and withdrawal reaction; problematic combination with MOR agonists | 3, 18, 19, 22 |
| central nervous system | Benzodiazepine Antagonist | flumazenil | seizures; individualisation of dosage | 4 |
| central nervous system | Catechol-O-Methyltransferase Inhibitor | tolcapone | hepatotoxity (potentially fatal acute liver failure, no retreatment in patient developed liver injury during treatment) | 1, 20 |
| central nervous system | Central alpha-2 Adrenergic Agonist | clonidine | risk of hemodynamic instability (hypotension, bradycardia) | 3 |
| central nervous system | Cholinesterase Inhibitor | pyridostigmine | pretreatment only for soman exposure (only benefit with use of atropine and 2-PAM, no use after exposure) | 20 |
| central nervous system | D2R-mGPCR antagonist | haloperidol | increased mortality in elderly patients with dementia-related psychosis | 17 |
| central nervous system | D2R-mGPCR antagonist | molindone | increased mortality in elderly patients with dementia-related psychosis | 17 |
| central nervous system | D2R-mGPCR antagonist | thiothixene | increased mortality in elderly patients with dementia-related psychosis | 17 |
| central nervous system | D2R-mGPCR antagonist | loxapine | increased mortality in elderly patients with dementia-related psychosis | 17 |
| central nervous system | Depolarizing Neuromuscular Blocker | succinylcholine | ventricular dysrhythmias, cardiac arrest, hyperkalemic rhandomyolysis | 3, 10, 15 |
| central nervous system | Dopamine Reuptake Inhibitor | modafinil | serious rash (SJS, TEN, DRESS); contraindicated in pediatric patients | 9, 14 |
| central nervous system | Dopamine-2 Receptor Antagonist | metoclopramide | tardive dyskinesia | 4 |
| central nervous system | GABA-enhancing Drugs | vigabatrin | permanent vision loss (bilateral concentric visual field constriction, tunnel vision) | 7 |
| central nervous system | Gamma-Aminobutyric Acid A Receptor Agonist | zaleplon | complex sleep behaviors (sleep-walking, sleep-driving, etc) | 4 |
| central nervous system | Gamma-Aminobutyric Acid A Receptor Agonist | zolpidem | complex sleep behaviors (sleep-walking, sleep-driving, etc) | 4 |
| central nervous system | Gamma-Aminobutyric Acid A Receptor Agonist | eszopiclone | complex sleep behaviors (sleep-walking, sleep-driving, etc) | 4 |
| central nervous system | Gamma-Aminobutyric Acid A Receptor Agonist | baclofen | no abrupt discontinuation (resulting in high fever, altered mental status, exaggerated rebound spasticity, muscle rigidity, rhandomyolysis, organ failure) | 20 |
| central nervous system | Glutamate Receptor Antagonist | felbamate | aplastic anaemia; hepatic failure | 1, 11 |
| central nervous system | Monoamine Oxidase Inhibitor | tranylcypromine | hypertensive crisis with significant tyramine ingestion; increased suicidality risk in children and young adults | 3, 12, 19 |
| central nervous system | Monoamine Oxidase Inhibitor | isocarboxazid | increased suicidality risk in children and young adults | 12 |
| central nervous system | Monoamine Oxidase Inhibitor | phenelzine sulfate | increased suicidality risk in children and young adults | 12 |
| central nervous system | Monoamine Oxidase Inhibitor | selegiline | increased suicidality risk in children and young adults | 12 |
| central nervous system | Monoamine Releasing Agent | amphetamine | abuse, misuse and addiction | 18 |
| ***Drug category*** | ***Drug class*** | ***Drug*** | ***Black box warning*** | ***Adverse reaction*** |
| central nervous system | Monoamine Reuptake Inhibitor | methylphenidate | abuse, misuse and addiction | 18 |
| central nervous system | MOR Agonist | remifentanil | addiction, abuse and misuse | 18 |
| central nervous system | MOR Agonist | sufentanil | addiction, abuse and misuse | 18 |
| central nervous system | MOR Agonist | alvimopan | potential risk of myocardial infarction with long-term use; for short-term hospital use and epidural administration only | 3 |
| central nervous system | MOR Agonist | propoxyphene | risk of fatal overdose (accidental and intentional); interaction with other CNS drugs and alcohol; drug interactions (CYP3A4) | 19 |
| central nervous system | MOR Agonist | hydromorphone | serious and life-threatening risks: addiction, abuse, misuse; respiratory depression; risk of combination with benzodiazepines and other CNS drugs; neonatal opioid withdrawal syndrome | 5, 18, 19 |
| central nervous system | MOR Agonist | levorphanol | serious and life-threatening risks: addiction, abuse, misuse; respiratory depression; risk of combination with benzodiazepines and other CNS drugs; neonatal opioid withdrawal syndrome | 5, 18, 19 |
| central nervous system | MOR Agonist | tapentadol | serious and life-threatening risks: addiction, abuse, misuse; respiratory depression; risk of combination with benzodiazepines and other CNS drugs; neonatal opioid withdrawal syndrome | 5, 18, 19 |
| central nervous system | MOR Agonist | pentazocine | serious and life-threatening risks: addiction, abuse, misuse; respiratory depression; risk of combination with benzodiazepines and other CNS drugs; neonatal opioid withdrawal syndrome | 5, 18, 19 |
| central nervous system | MOR Agonist | buprenorphine | serious and life-threatening risks: addiction, abuse, misuse; respiratory depression; risk of combination with benzodiazepines and other CNS drugs; neonatal opioid withdrawal syndrome | 5, 18, 19 |
| central nervous system | MOR Agonist | butorphanol | serious and life-threatening risks: addiction, abuse, misuse; respiratory depression; risk of combination with benzodiazepines and other CNS drugs; neonatal opioid withdrawal syndrome; drug interactions (CYP3A4) | 5, 18, 19 |
| central nervous system | MOR Agonist | codeine | serious and life-threatening risks: addiction, abuse, misuse; respiratory depression; risk of combination with benzodiazepines and other CNS drugs; neonatal opioid withdrawal syndrome; drug interactions (CYP3A4) | 5, 18, 19 |
| central nervous system | MOR Agonist | hydrocodone | serious and life-threatening risks: addiction, abuse, misuse; respiratory depression; risk of combination with benzodiazepines and other CNS drugs; neonatal opioid withdrawal syndrome; drug interactions (CYP3A4) | 5, 18, 19 |
| central nervous system | MOR Agonist | oxycodone | serious and life-threatening risks: addiction, abuse, misuse; respiratory depression; risk of combination with benzodiazepines and other CNS drugs; neonatal opioid withdrawal syndrome; drug interactions (CYP3A4) | 5, 18, 19 |
| central nervous system | MOR Agonist | fentanyl | serious and life-threatening risks: addiction, abuse, misuse; respiratory depression; risk of combination with benzodiazepines and other CNS drugs; neonatal opioid withdrawal syndrome; drug interactions (CYP3A4); risk of medication errors | 5, 18, 19 |
| central nervous system | MOR Agonist | oxymorphone | serious and life-threatening risks: addiction, abuse, misuse; respiratory depression; risk of combination with benzodiazepines and other CNS drugs; neonatal opioid withdrawal syndrome; interaction with alcohol | 5, 18, 19 |
| central nervous system | MOR Agonist | methadone | serious and life-threatening risks: addiction, abuse, misuse; respiratory depression; risk of combination with benzodiazepines and other CNS drugs; neonatal opioid withdrawal syndrome; QT interval prolongation and torsades de pointes | 3, 5, 18, 19 |
| ***Drug category*** | ***Drug class*** | ***Drug*** | ***Black box warning*** | ***Adverse reaction*** |
| central nervous system | MOR Agonist | tramadol | serious and life-threatening risks: addiction, abuse, misuse; respiratory depression; risk of combination with benzodiazepines and other CNS drugs; neonatal opioid withdrawal syndrome; ultra-rapid metabolizer due to CYP2D6 polymorphism; drug interactions (cytochrome P450 3A4 or 2D6) | 5, 18, 19 |
| central nervous system | MOR Agonist | morphine | serious and life-threatening risks: respiratory depression; risk of combination with benzodiazepines and other CNS drugs | 5, 19 |
| central nervous system | MOR Agonist | nalbuphine | serious and life-threatening risks: respiratory depression; risk of combination with benzodiazepines and other CNS drugs | 5, 19 |
| central nervous system | MOR Antagonist | naltrexone | hepatotoxicity (excessive doses, active liver disease) | 1, 20 |
| central nervous system | Neuroactive Steroid Gamma-Aminobutyric Acid A Receptor Positive Modulator | zuranolone | impaired ability to drive (CNS depressant effects) | 4 |
| central nervous system | Neurokinin 3 Receptor Antagonist | fezolinetant | hepatotoxicity | 1 |
| central nervous system | Noncompetitive AMPA Glutamate Receptor Antagonist | esketamine | sedation; dissociation; suicidal thoughts and behaviours; abuse and misuse; respiratory depression | 4, 5, 12, 18 |
| central nervous system | Noncompetitive AMPA Glutamate Receptor Antagonist | perampanel | serious psychiatric and behavioural reactions (aggression, hostility, irritability, anger, homicidal ideations) | 12 |
| central nervous system | Nondepolarizing Neuromuscular Blocker | vecuronium | administration by adequately trained personnel | 22 |
| central nervous system | Non-narcotic Antitussive | benzonatate | hypersensitivity reactions (bronchospasm, laryngospasm, cardiovascular collapse); psychiatric effects (bizarre behavior, mental confusion, visual hallucinations), accidental ingestion | 12, 14 |
| central nervous system | Non-selective Serotonin and Norepinephrine Reuptake Inhibitor | amitriptyline | increased suicidality risk in children and young adults | 12 |
| central nervous system | Non-selective Serotonin and Norepinephrine Reuptake Inhibitor | clomipramine | increased suicidality risk in children and young adults | 12 |
| central nervous system | Non-selective Serotonin and Norepinephrine Reuptake Inhibitor | desipramine | increased suicidality risk in children and young adults | 12 |
| central nervous system | Non-selective Serotonin and Norepinephrine Reuptake Inhibitor | doxepin | increased suicidality risk in children and young adults | 12 |
| central nervous system | Non-selective Serotonin and Norepinephrine Reuptake Inhibitor | imipramine | increased suicidality risk in children and young adults | 12 |
| central nervous system | Non-selective Serotonin and Norepinephrine Reuptake Inhibitor | nortriptyline | increased suicidality risk in children and young adults | 12 |
| central nervous system | Non-selective Serotonin and Norepinephrine Reuptake Inhibitor | protriptyline | increased suicidality risk in children and young adults | 12 |
| central nervous system | Non-selective Serotonin and Norepinephrine Reuptake Inhibitor | trimipramine | increased suicidality risk in children and young adults | 12 |
| central nervous system | Norepinephrine Reuptake Inhibitor | atomoxetine | suicidal ideation in children and adolescents | 12 |
| central nervous system | Norepinephrine Reuptake Inhibitor | violoxazine | suicidal thoughts and behaviours | 12 |
| central nervous system | not specified | acetaminophen | hepatotoxicity (liver failure in overdose) | 1 |
| central nervous system | N-type Calcium Channel Antagonist | ziconotide | neuropsychiatric adverse reactions (psychiatric symptoms and neurological impairment, contraindicated in psychosis) | 12 |
| ***Drug category*** | ***Drug class*** | ***Drug*** | ***Black box warning*** | ***Adverse reaction*** |
| central nervous system | Phenothiazine | fluphenazine | increased mortality in elderly patients with dementia-related psychosis | 17 |
| central nervous system | Phenothiazine | perphenazine | increased mortality in elderly patients with dementia-related psychosis | 17 |
| central nervous system | Phenothiazine | prochlorperazine | increased mortality in elderly patients with dementia-related psychosis | 17 |
| central nervous system | Phenothiazine | trifluoperazine | increased mortality in elderly patients with dementia-related psychosis | 17 |
| central nervous system | Phenothiazine | chlorpromazine | increased mortality in elderly patients with dementia-related psychosis | 17 |
| central nervous system | Phenothiazine | thioridazine | increased mortality in elderly patients with dementia-related psychosis; prolonged QTc interval and Torsades de pointes arrhythmia | 3, 17 |
| central nervous system | Phenothiazine | dextromethorphan | respiratory depression (contraindicated in age below 2 years) | 5 |
| central nervous system | Phenothiazine | promethazine | respiratory depression (contraindicated in age below 2 years) | 5 |
| central nervous system | p-mGPCR antagonist | iloperidone | increased mortality in elderly patients with dementia-related psychosis | 17 |
| central nervous system | p-mGPCR antagonist | olanzapine | increased mortality in elderly patients with dementia-related psychosis | 17 |
| central nervous system | p-mGPCR antagonist | paliperidone | increased mortality in elderly patients with dementia-related psychosis | 17 |
| central nervous system | p-mGPCR antagonist | pimavanserin tartrate | increased mortality in elderly patients with dementia-related psychosis | 17 |
| central nervous system | p-mGPCR antagonist | quetiapine | increased mortality in elderly patients with dementia-related psychosis | 17 |
| central nervous system | p-mGPCR antagonist | risperidone | increased mortality in elderly patients with dementia-related psychosis | 17 |
| central nervous system | p-mGPCR antagonist | ziprasidone | increased mortality in elderly patients with dementia-related psychosis | 17 |
| central nervous system | p-mGPCR antagonist | aripiprazole | increased mortality in elderly patients with dementia-related psychosis; increased suicidality risk in children and young adults | 12, 17 |
| central nervous system | p-mGPCR antagonist | brexpiprazole | increased mortality in elderly patients with dementia-related psychosis; increased suicidality risk in children and young adults | 12, 17 |
| central nervous system | p-mGPCR antagonist | cariprazine | increased mortality in elderly patients with dementia-related psychosis; increased suicidality risk in children and young adults | 12, 17 |
| central nervous system | p-mGPCR antagonist | lumateperone | increased mortality in elderly patients with dementia-related psychosis; increased suicidality risk in children and young adults | 12, 17 |
| central nervous system | p-mGPCR antagonist | lurasidone | increased mortality in elderly patients with dementia-related psychosis; increased suicidality risk in children and young adults | 12, 17 |
| central nervous system | p-mGPCR antagonist | clozapine | severe neutropenia; orthostatic hypotension, bradycardia and syncope; seizure; myocarditis, pericarditis and cardiomyopathy; increased mortalitiy in elderly patients with dementia-related psychosis | 3, 4, 11, 17, 21 |
| central nervous system | Ryanodine Receptor 1 Antagonist | dantrolene | hepatotoxicity (symptomatic hepatitis, serious hepatic injury, liver enzyme elevations) | 1 |
| central nervous system | Serotonin 5-HT2 agonist | fenfluramine | valvular heart disease; pulmonary arterial hypertension | 3, 5 |
| central nervous system | Serotonin and Norepinephrine Reuptake Inhibitor | milnacipran | increased suicidality risk in children and young adults | 12 |
| central nervous system | Serotonin and Norepinephrine Reuptake Inhibitor | desvenlafaxine | suicidal thoughts and behaviours | 12 |
| central nervous system | Serotonin and Norepinephrine Reuptake Inhibitor | duloxetine | suicidal thoughts and behaviours | 12 |
| ***Drug category*** | ***Drug class*** | ***Drug*** | ***Black box warning*** | ***Adverse reaction*** |
| central nervous system | Serotonin and Norepinephrine Reuptake Inhibitor | levomilnacipran | suicidal thoughts and behaviours | 12 |
| central nervous system | Serotonin and Norepinephrine Reuptake Inhibitor | venlafaxine | suicidal thoughts and behaviours | 12 |
| central nervous system | Serotonin Receptor Agonist | ergotamine | peripheral ischemia following co-administration with potent CYP3A4 inhibitors | 19 |
| central nervous system | Serotonin Reuptake Inhibitor | fluvoxamine | increased suicidality risk in children and young adults | 12 |
| central nervous system | Serotonin Reuptake Inhibitor | nefazodone | increased suicidality risk in children and young adults | 12 |
| central nervous system | Serotonin Reuptake Inhibitor | citalopram | suicidal thoughts and behaviours | 12 |
| central nervous system | Serotonin Reuptake Inhibitor | escitalopram | suicidal thoughts and behaviours | 12 |
| central nervous system | Serotonin Reuptake Inhibitor | fluoxetine | suicidal thoughts and behaviours | 12 |
| central nervous system | Serotonin Reuptake Inhibitor | paroxetine | suicidal thoughts and behaviours | 12 |
| central nervous system | Serotonin Reuptake Inhibitor | sertraline | suicidal thoughts and behaviours | 12 |
| central nervous system | Serotonin Reuptake Inhibitor | trazodone | suicidal thoughts and behaviours | 12 |
| central nervous system | Serotonin Reuptake Inhibitor | gepirone | suicidal thoughts and behaviours | 12 |
| central nervous system | Serotonin Reuptake Inhibitor | vilazodone | suicidal thoughts and behaviours | 12 |
| central nervous system | Serotonin Reuptake Inhibitor | vortioxetine | suicidal thoughts and behaviours | 12 |
| central nervous system | Serotonin-1b and Serotonin-1d Receptor Agonist | flibanserin | hypotension and syncope in certain settings (particularly hepatic impairment); interaction with alcohol; drug interactions (contraindicated with CYP3A4 inhibitors) | 3, 19, 20 |
| central nervous system | Serotonin-1b and Serotonin-1d Receptor Agonist | rizatriptan | limitations of use (only in established diagnosis) | 22 |
| central nervous system | Serotonin-3 Receptor Antagonist | alosetron | serious gastrointestinal adverse reactions (ischemic colitits, constipation) | 8 |
| central nervous system | Sodium Channel Blocker | cocaine | abuse and dependence | 18 |
| central nervous system | Sodium Channel Blocker | bupivacaine | cardiac arrest (systemic toxicity following unintentional intravascular injection); not recommended in obstretical anesthesia | 3 |
| central nervous system | Sodium Channel Blocker | phenytoin | cardiovascular risk in rapid infusion; cardiac monitoring necessary | 3, 22 |
| central nervous system | Sodium Channel Blocker | valproic acid | hepatotoxicity; embryo-fetal toxicityity (neural tube defects, major malformations, decreased IQ); pancreatitis | 1, 13, 15 |
| central nervous system | Sodium Channel Blocker | lidocaine | life-threatening events in children (seizures, cardiopulmonary arrest); limitation of use (not for teething pain in children) | 3, 4, 5, 17 |
| central nervous system | Sodium Channel Blocker | carbamazepine | serious dermatologic reactions (toxic epidermal necrolysis, stevens-johnson syndrome, particularly in HLA-B*1502 allele); aplastic anemia and agranulocytosis | 9, 11, 14 |
| central nervous system | Sodium Channel Blocker | lamotrigine | serious skin rashes (SJS, TEN) | 9, 14 |
| central nervous system | Vesicular Monoamine Transporter 2 Inhibitor | tetrabenazine | risk of depression and suicidal thoughts in patients with Huntington’s disease | 12 |
| central nervous system | Vesicular Monoamine Transporter 2 Inhibitor | valbenazine | risk of depression and suicidal thoughts in patients with Huntington’s disease | 12 |
| ***Drug category*** | ***Drug class*** | ***Drug*** | ***Black box warning*** | ***Adverse reaction*** |
| central nervous system | Vesicular Monoamine Transporter 2 Inhibitor | deutetrabenazine | risk of depression and suicidal thoughts in patients with Huntington’s disease | 12 |
| cytotoxic treatment | Alkylating Drug | lomustine | delayed myelosuppression (particularly thrombocytopenia); risk of overdosage (fatal toxicity) | 11 |
| cytotoxic treatment | Alkylating Drug | busulfan | myelosuppression | 11 |
| cytotoxic treatment | Alkylating Drug | treosulfan | myelosuppression | 11 |
| cytotoxic treatment | Alkylating Drug | chlorambucil | myelosuppression (thrombocytopenia, leukopenia); pulmonary toxicity (dose-related) | 5, 11 |
| cytotoxic treatment | Alkylating Drug | carmustine | myelosuppression (thrombocytopenia, leukopenia); pulmonary toxicity (dose-related) | 5, 11 |
| cytotoxic treatment | Alkylating Drug | temozolomide | myelosuppression; myelodysplastic syndrome and secondary malignancies (myeloid leukemia); pneumocystis carinii pneumonia prophylaxis required; teratogenic effect | 11, 13, 16, 21 |
| cytotoxic treatment | Alkylating Drug | ifosfamide | myelosuppression; neurotoxicity (encephalopathy); urotoxicity (renal failure) | 2, 4, 11 |
| cytotoxic treatment | Alkylating Drug | thiotepa | severe myelosuppression; carcinogenicity | 11, 16 |
| cytotoxic treatment | Alkylating Drug | procarbazine | supervision of trained physician; availability of facilities for monitoring of treatment | 22 |
| cytotoxic treatment | Alkylating Drug | dacarbazine | supervision of trained professional; hematopoietic depression; hepatic necrosis; carcinogenic and teratogenic effect | 1, 11, 13, 16, 22 |
| cytotoxic treatment | Alkylating Drug | mitomycin | supervision of trained professional; myelosuppression (thrombocytopenia, leukopenia); hemolytic uremic syndrome | 2, 11, 22 |
| cytotoxic treatment | Alkylating Drug | melphalan | supervision of trained professional; myelosuppression; hypersensitivity reactions (anaphylaxis); hepatic necrosis; leukemogenic and mutagenic | 1, 11, 13, 14, 16, 22 |
| cytotoxic treatment | Anthracycline Topoisomerase Inhibitor | doxorubicin | supervision of trained physician; severe local tissue necrosis in extravasation; myocardial toxicity; secondary AML or myelodysplastic syndrome; severe myelosuppression; dosage adjustmentt in impaired hepatic function | 3, 9, 11, 22 |
| cytotoxic treatment | Anthracycline Topoisomerase Inhibitor | epirubicin | supervision of trained physician; severe local tissue necrosis in extravasation; myocardial toxicity; secondary AML; severe myelosuppression; dosage adjustmentt in impaired hepatic function | 3, 9, 11, 22 |
| cytotoxic treatment | Anthracycline Topoisomerase Inhibitor | idarubicin | supervision of trained physician; severe local tissue necrosis in extravasation; myocardial toxicity; severe myelosuppression; dosage adjustmentt in impaired hepatic function | 3, 9, 11, 22 |
| cytotoxic treatment | Anthracycline Topoisomerase Inhibitor | daunorubicin | supervision of trained physician; severe myelosuppression; myocardial toxicity; severe local tissue necrosis in extravasation | 3, 9, 11, 22 |
| cytotoxic treatment | Antimetabolite | hydroxyurea | myelosuppression; carcinogenic | 11, 16 |
| cytotoxic treatment | Antimetabolite | floxuridine | supervision of trained physician; severe toxic reactions; need of hospitalization for first course of therapy | 22 |
| cytotoxic treatment | Antisense Oligonucleotide | inotersen | thrombocytopenia; glomerulonephritis | 2, 11 |
| cytotoxic treatment | Arsenic Compound | arsenic cation (3+) | differentiation syndrome; cardiac conduction abnormalities (QTc interval prolongation, atrioventricular block, torsade de pointes); encephalopathy (including Wernicke’s) | 3, 4, 14 |
| cytotoxic treatment | CD123 Interaction | tagraxofusp | capillary leak syndrome | 3 |
| cytotoxic treatment | Collagen-specific Enzyme | collagenase clostridium histolyticum | penile injury (corporal rupture, hematoma) | 3 |
| ***Drug category*** | ***Drug class*** | ***Drug*** | ***Black box warning*** | ***Adverse reaction*** |
| cytotoxic treatment | Epidermal Growth Factor Receptor Antagonist | necitumumab | cardiopulmonary arrest; hypomagnesemia | 3, 5, 15 |
| cytotoxic treatment | Epidermal Growth Factor Receptor Antagonist | panitumumab | dermatologic toxicity | 9 |
| cytotoxic treatment | Epidermal Growth Factor Receptor Antagonist | cetuximab | infusion reaction; cardiopulmonary arrest | 3, 5, 14 |
| cytotoxic treatment | Folate Analog Metabolic Inhibitor | methotrexate | embryo-fetal toxicity; hypersensitivity reaction; benzyl alcohol toxicity (central nervous toxicity, metabolic acidosis); other adverse reactions (bone marrow, kidneys, liver, nervous system, gastrointestinal tract, lungs, skin) | 1, 2, 4, 5, 8, 9, 11, 13, 15 |
| cytotoxic treatment | Glycopeptide Antibacterial | bleomycin | supervision of trained professional; pulmonary fibrosis; idiosyncratic reaction (hypotension, mental confusion, fever, chill, wheezing) | 5, 11, 22 |
| cytotoxic treatment | Hedgehog Pathway Inhibitor | glasdegib | embryo-fetal toxicity | 13 |
| cytotoxic treatment | Hedgehog Pathway Inhibitor | sonidegib | embryo-fetal toxicity | 13 |
| cytotoxic treatment | Hedgehog Pathway Inhibitor | vismodegib | embryo-fetal toxicity | 13 |
| cytotoxic treatment | HER2/neu Receptor Antagonist | trastuzumab | embryo-fetal toxicity; cardiomyopathy; pulmonary toxicity | 3, 5, 13 |
| cytotoxic treatment | HER2/neu Receptor Antagonist | pertuzumab | embryo-fetal toxicity; left ventricular dysfunction | 3, 13 |
| cytotoxic treatment | Histone Deacetylase Inhibitor | panobinostat | severe diarrhea; cardiac toxicity (cardiac ischemic events, arrhythmia, ECG changes) | 5, 8 |
| cytotoxic treatment | Hypoxia-inducible Factor Inhibitor | belzutifan | embryo-fetal toxicityity | 13 |
| cytotoxic treatment | Isocitrate Dehydrogenase 1 Inhibitor | ivosidenib | differentiation syndrome | 14 |
| cytotoxic treatment | Isocitrate Dehydrogenase 2 Inhibitor | enasidenib | differentiation syndrome | 14 |
| cytotoxic treatment | Janus Kinase Inhibitor | fedratinib | encephalopathy (including Wernicke’s) | 4 |
| cytotoxic treatment | Janus Kinase Inhibitor | baricitinib | serious infections (particularly active tuberculosis, invasive fungal infections, opportunistic pathogens); increased all-cause mortality; malignancies; major adverse cardiovascular events; thrombosis (deep venous thrombosis, pulmonary embolism, arterial thrombosis) | 3, 11, 16, 17, 21 |
| cytotoxic treatment | Janus Kinase Inhibitor | deuruxolitinib | serious infections (particularly active tuberculosis, invasive fungal infections, opportunistic pathogens); increased all-cause mortality; malignancies; major adverse cardiovascular events; thrombosis (deep venous thrombosis, pulmonary embolism, arterial thrombosis) | 3, 11, 16, 17, 21 |
| cytotoxic treatment | Janus Kinase Inhibitor | ruxolitinib | serious infections (particularly active tuberculosis, invasive fungal infections, opportunistic pathogens); increased all-cause mortality; malignancies; major adverse cardiovascular events; thrombosis (deep venous thrombosis, pulmonary embolism, arterial thrombosis) | 3, 11, 16, 17, 21 |
| cytotoxic treatment | Janus Kinase Inhibitor | tofacitinib | serious infections (particularly active tuberculosis, invasive fungal infections, opportunistic pathogens); increased all-cause mortality; malignancies; major adverse cardiovascular events; thrombosis (deep venous thrombosis, pulmonary embolism, arterial thrombosis) | 3, 11, 16, 17, 21 |
| ***Drug category*** | ***Drug class*** | ***Drug*** | ***Black box warning*** | ***Adverse reaction*** |
| cytotoxic treatment | Janus Kinase Inhibitor | upadacitinib | serious infections (particularly active tuberculosis, invasive fungal infections, opportunistic pathogens); increased all-cause mortality; malignancies; major adverse cardiovascular events; thrombosis (deep venous thrombosis, pulmonary embolism, arterial thrombosis) | 3, 11, 16, 17, 21 |
| cytotoxic treatment | Janus Kinase Inhibitor | ritlecitinib | serious infections (particularly active tuberculosis, invasive fungal infections, opportunistic pathogens); increased all-cause mortality; malignancies; major adverse cardiovascular events; thrombosis (deep venous thrombosis, pulmonary embolism, arterial thrombosis) | 3, 11, 16, 17, 21 |
| cytotoxic treatment | Janus Kinase Inhibitor | abrocitinib | serious infections (particularly herpes simplex, herpes zoster, pneumonia); increased all-cause mortality; malignancies; major adverse cardiovascular events; thrombosis (deep venous thrombosis, pulmonary embolism, arterial thrombosis) | 3, 11, 17, 21 |
| cytotoxic treatment | Kinase Inhibitor | ponatinib | arterial occlusive events (myocardial infarction, stroke, stenosis of arterial vessels, peripheral vascular disease); venous thromboembolic events; heart failure; hepatotoxicity | 1, 3 |
| cytotoxic treatment | Kinase Inhibitor | gilteritinib | differentiation syndrome | 3 |
| cytotoxic treatment | Kinase Inhibitor | idelalisib | fatal and serious toxicities: hepatotoxicity; diarrhea; colitis; pneumonitis; infections; intestinal perforation | 1, 5, 8, 17, 21 |
| cytotoxic treatment | Kinase Inhibitor | lapatinib | hepatotoxicity | 1 |
| cytotoxic treatment | Kinase Inhibitor | pazopanib | hepatotoxicity | 1 |
| cytotoxic treatment | Kinase Inhibitor | pexidartinib | hepatotoxicity | 1 |
| cytotoxic treatment | Kinase Inhibitor | regorafenib | hepatotoxicity | 1 |
| cytotoxic treatment | Kinase Inhibitor | sunitinib | hepatotoxicity | 1 |
| cytotoxic treatment | Kinase Inhibitor | nilotinib | QT prolongation; sudden deaths | 3, 17 |
| cytotoxic treatment | Kinase Inhibitor | quizartinib | QT prolongation; torsades de points; cardiac arrest | 3 |
| cytotoxic treatment | Kinase Inhibitor | vandetanib | QT prolongation; torsades de points; sudden death | 3, 17 |
| cytotoxic treatment | Kinase Inhibitor | duvelisib | treatment-related mortality and serious toxicities: infections; diarrhea; colitis; cutaneous reaction; pneumonitis | 5, 8, 9, 17, 21 |
| cytotoxic treatment | Lysosomal beta Glucuronidase | vestronidase alfa | anaphylaxis | 14 |
| cytotoxic treatment | Menin Inhibitor | revumenib | differentiation syndrome; QTc prolongation; torsades de pointes | 3 |
| cytotoxic treatment | Microtubule Inhibitor | cabazitaxel | neutropenia; hypersensitivity reactions | 11, 14 |
| cytotoxic treatment | Microtubule Inhibitor | paclitaxel | supervision of trained professional; hypersensitivity reactions (anaphylaxis, dyspnea, hypotension, angioedema, urticaria); bone marrow suppression (neutropenia) | 11, 14, 22 |
| cytotoxic treatment | Microtubule Inhibitor | docetaxel | toxic deaths (increased treatment-related mortality); hepatotoxicity; neutropenia; hypersensitivity reactions (rash, hypotension, bronchospasm, anaphylaxis); fluid retention | 1, 11, 14, 15, 17 |
| cytotoxic treatment | Nucleoside Metabolic Inhibitor | nelarabine | neurologic adverse reactions (altered mental states, severe somnolence, convulsions, peripheral neuropathy, demyelination) | 4 |
| cytotoxic treatment | Nucleoside Metabolic Inhibitor | fluorouracil | serious adverse reactions in patients with complete DPD deficiency | 19 |
| ***Drug category*** | ***Drug class*** | ***Drug*** | ***Black box warning*** | ***Adverse reaction*** |
| cytotoxic treatment | Nucleoside Metabolic Inhibitor | cytarabine | supervision of trained professional; availability of necessary facilities | 22 |
| cytotoxic treatment | Nucleoside Metabolic Inhibitor | fludarabine | supervision of trained professional; availability of necessary facilities; autoimmune hemolytic anemia; no combination with pentostatin (pulmonary toxicity) | 14, 19, 22 |
| cytotoxic treatment | Nucleoside Metabolic Inhibitor | pentostatin | supervision of trained professional; no combination with fludarabine phosphate (pulmonary toxicity) | 19, 22 |
| cytotoxic treatment | Nucleoside Metabolic Inhibitor | capecitabine | warfarin interaction (bleeding) | 19 |
| cytotoxic treatment | Photoactivated Radical Generator | methoxsalen | supervision of trained professional; restricted use to severe and treatment-refractory psoriasis; ocular damage; skin agin; skin cancer | 7, 9, 16, 22 |
| cytotoxic treatment | Platinum-based Drug | oxaliplatin | anaphylaxis | 14 |
| cytotoxic treatment | Platinum-based Drug | carboplatin | supervision of trained professional; availability of necessary facilities; bone marrow suppression; anaphylactic-like reactions | 11, 14, 22 |
| cytotoxic treatment | Platinum-based Drug | cisplatin | supervision of trained professional; availability of necessary facilities; renal toxicity (cumulative); myelosuppression; ototoxicity; anaphylactic-like reaction | 2, 6, 11, 14, 22 |
| cytotoxic treatment | Purine Antimetabolite | cladribine | supervision of trained professional; bone marrow suppression; neurological toxicity (irreversible paraparesis, quadriparesis); nephrotoxicity | 2, 4, 11, 22 |
| cytotoxic treatment | Pyrimidine Synthesis Inhibitor | teriflunomide | embryo-fetal toxicityity; hepatotoxicity | 1, 13 |
| cytotoxic treatment | Topoisomerase Inhibitor | topotecan | myelosuppression | 11 |
| cytotoxic treatment | Topoisomerase Inhibitor | irinotecan | myelosuppression; diarrhea (early and late forms, cholinergic symptoms) | 8, 11 |
| cytotoxic treatment | Topoisomerase Inhibitor | mitoxantrone | supervision of trained professional; local tissue damage in extravasation; bone marrow suppression (neutropenia); cardiotoxicity; malignancies (secondary leukemia) | 3, 9, 11, 16, 22 |
| cytotoxic treatment | Topoisomerase Inhibitor | etoposide | supervision of trained professional; myelosuppression | 11, 22 |
| cytotoxic treatment | Vinca Alkaloid | vinblastine | leakage in surrounding tissue resulting in considerable irritation; for intravenous use only (fatal if given by other routes) | 9 |
| cytotoxic treatment | Vinca Alkaloid | vinorelbine | myelosuppression (serious infection, septic shock); dosage adjustmentts in special populations | 11 |
| cytotoxic treatment | Vinca Alkaloid | vincristine | supervision of trained professional; local tissue damage in extravasation; for intravenous use only (fatal in other routes) | 9, 22 |
| cytotoxic treatment | Viral DNA Polymerase Inhibitor | foscarnet | nephrotoxicty; seizures | 2, 4 |
| diagnostic drugs | Contrast Agent for Ultrasound Imaging | perflutren | cardiopulmonary reactions; supervision of trained professional and availability of adequate facilites | 3, 22 |
| diagnostic drugs | Non-Standardized Animal Skin Allergenic Extract | cat dander, dog dander, horse dander, cattle dander, sheep dander, goat dander, guinea pig dander, rabbit dander, mouse dander, rat dander | anaphylaxis; supervision of trained professional | 14, 22 |
| diagnostic drugs | Non-Standardized Bacterial Allergenic Extract | streptococcus, staphylococcus, pneumococcus, proteus, escherichia coli, klebsiella | anaphylaxis; supervision of trained professional | 14, 22 |
| ***Drug category*** | ***Drug class*** | ***Drug*** | ***Black box warning*** | ***Adverse reaction*** |
| diagnostic drugs | Non-Standardized Chemical Allergen | formaldehyde, nickel, chromium, cobalt, latex chemicals, epoxy resin, rubber accelerators | anaphylaxis; supervision of trained professional | 14, 22 |
| diagnostic drugs | Non-Standardized Chemical Allergen | lindane | anaphylaxis; supervision of trained professional | 14, 22 |
| diagnostic drugs | Non-Standardized Feather Allergenic Extract | chicken feather, duck feather, goose feather, turkey feather, mixed bird feather | anaphylaxis; supervision of trained professional | 14, 22 |
| diagnostic drugs | Non-Standardized Food Allergenic Extract | beef, pork, chicken, turkey, egg, egg white, egg yolk, cow milk, goat milk, cheese, wheat, rye, barley, oat, rice, corn, soy, peanut, almond, walnut, pecan, hazelnut, cashew, pistachio, sesame, fish, shellfish, shrimp, crab, lobster, tuna, cod, salmon, apple, banana, orange, strawberry, tomato, potato, carrot, celery, onion, garlic, cocoa, coffee | anaphylaxis; supervision of trained professional | 14, 22 |
| diagnostic drugs | Non-Standardized Fungal Allergenic Extract | alternaria, aspergillus, cladosporium, epicoccum, fusarium, helminthosporium, mucor, penicillium, rhizopus, trichophyton, candida | anaphylaxis; supervision of trained professional | 14, 22 |
| diagnostic drugs | Non-Standardized House Dust Allergenic Extract | house dust, dust mite, storage mite | anaphylaxis; supervision of trained professional | 14, 22 |
| diagnostic drugs | Non-Standardized Insect Allergenic Extract | cockroach, beetle, moth, fly, mosquito, locust | anaphylaxis; supervision of trained professional | 14, 22 |
| diagnostic drugs | Non-Standardized Insect Venom Allergenic Extract | bee venom, honeybee venom, wasp venom, yellow jacket venom, hornet venom, fire ant venom | anaphylaxis; supervision of trained professional | 14, 22 |
| diagnostic drugs | Non-Standardized Plant Allergenic Extract | cotton, hemp, flax, tobacco, hops, alfalfa, clover | anaphylaxis; supervision of trained professional | 14, 22 |
| diagnostic drugs | Non-Standardized Plant Fiber Allergenic Extract | cotton fiber, kapok fiber, flax fiber, jute fiber, sisal fiber | anaphylaxis; supervision of trained professional | 14, 22 |
| diagnostic drugs | Non-Standardized Plant Fiber Allergenic Extract | grain mill dust | anaphylaxis; supervision of trained professional | 14, 22 |
| ***Drug category*** | ***Drug class*** | ***Drug*** | ***Black box warning*** | ***Adverse reaction*** |
| diagnostic drugs | Non-Standardized Pollen Allergenic Extract | acacia, alder, ash, birch, cedar, cottonwood, cypress, elm, eucalyptus, hackberry, hickory, juniper, maple, mulberry, oak, olive, pecan, pine, plane tree, poplar, redwood, sweetgum, walnut, bermuda grass, bluegrass, brome grass, canary grass, fescue, johnson grass, orchard grass, oat, rye grass, timothy grass, wheatgrass, amaranth, sagebrush, mugwort, pigweed, ragweed, cocklebur, lamb’s quarters, plantain, russian thistle, saltbush, nettle, dandelion, dock | anaphylaxis; supervision of trained professional | 14, 22 |
| diagnostic drugs | Paramagnetic Contrast Agent | pegulicianine | anaphylaxis; supervision of trained professional | 14, 22 |
| diagnostic drugs | Paramagnetic Contrast Agent | Gadolinium Cation (3+) | nephrogenic systemic fibrosis in impaired renal function; risk associated with intrathecal use and not approved (coma, encephalopathy, seizures) | 2, 4 |
| diagnostic drugs | Paramagnetic Contrast Agent | gadopiclenol | nephrogenic systemic fibrosis in impaired renal function; risk associated with intrathecal use and not approved (coma, encephalopathy, seizures) | 2, 4 |
| diagnostic drugs | Paramagnetic Contrast Agent | gadodiamide | nephrogenic systemic fibrosis in impaired renal function; risk associated with intrathecal use and not approved (coma, encephalopathy, seizures) | 2, 4 |
| diagnostic drugs | Paramagnetic Contrast Agent | gadoteridol | nephrogenic systemic fibrosis in impaired renal function; risk associated with intrathecal use and not approved (coma, encephalopathy, seizures) | 2, 4 |
| diagnostic drugs | Radioactive Diagnostic Agent | rubidium cation | high radiation exposure in incorrect use | 22 |
| diagnostic drugs | Radioactive Diagnostic Agent | gallium cation | nephrotoxicity in patients with cancer-related hypercalcemia (particularly in combination with other nephrotoxic drugs) | 2, 19 |
| diagnostic drugs | Radioactive Diagnostic Agent | iodixanol | risk associated with intrathecal use and not approved (coma, encephalopathy, seizures, renal failure, cardiac arrest, rhandomyolysis, hyperthermia, brain edema) | 2, 3, 4, 10, 15 |
| diagnostic drugs | Radiographic Contrast Agent | gadofosceset | nephrogenic systemic fibrosis in impaired renal function | 2 |
| diagnostic drugs | Radiographic Contrast Agent | iopamidol | risk associated with intrathecal use and not approved (coma, encephalopathy, seizures, renal failure, cardiac arrest, rhandomyolysis, hyperthermia, brain edema) | 2, 3, 4, 10, 15 |
| diagnostic drugs | Radiographic Contrast Agent | iopromide | risk associated with intrathecal use and not approved (coma, encephalopathy, seizures, renal failure, cardiac arrest, rhandomyolysis, hyperthermia, brain edema) | 2, 3, 4, 10, 15 |
| diagnostic drugs | Radiographic Contrast Agent | ioversol | risk associated with intrathecal use and not approved (coma, encephalopathy, seizures, renal failure, cardiac arrest, rhandomyolysis, hyperthermia, brain edema) | 2, 3, 4, 10, 15 |
| diagnostic drugs | Radiographic Contrast Agent | iohexol | risk associated with wrong iodine concentration for application (coma, encephalopathy, seizures, renal failure, cardiac arrest, rhandomyolysis, hyperthermia, brain edema) | 2, 3, 4, 10, 15 |
| ***Drug category*** | ***Drug class*** | ***Drug*** | ***Black box warning*** | ***Adverse reaction*** |
| diagnostic drugs | Skin Test Antigen | coccidioides immitis spherule-derived skin test antighen | anaphylaxis; supervision of trained professional | 14, 22 |
| diagnostic drugs | Skin Test Antigen | tenapanor | risk of dehydration in children (contraindicated in age below 6 years) | 15 |
| diagnostic drugs | Standardized Animal Hair Allergenic Extract | cat hair | anaphylaxis; supervision of trained professional | 14, 22 |
| diagnostic drugs | Standardized Animal Hair Allergenic Extract | cat hair, dog hair | anaphylaxis; supervision of trained professional | 14, 22 |
| diagnostic drugs | Standardized Animal Skin Allergenic Extract | cat dander, cat skin | anaphylaxis; supervision of trained professional | 14, 22 |
| diagnostic drugs | Standardized Insect Allergenic Extract | dust mite (Dermatophagoides farinae), dust mite (Dermatophagoides pteronyssinus) | anaphylaxis; supervision of trained professional | 14, 22 |
| diagnostic drugs | Standardized Insect Venom Allergenic Extract | honeybee venom, yellow hornet venom, white-faced hornet venom, paper wasp venom, German yellow jacket venom, eastern yellow jacket venom, western yellow jacket venom, southern yellow jacket venom, common yellow jacket venom | anaphylaxis; supervision of trained professional | 14, 22 |
| diagnostic drugs | Standardized Pollen Allergenic Extract | ragweed, timothy grass, bermuda grass, orchard grass, perennial rye grass, june grass, oak pollen, birch pollen | anaphylaxis; supervision of trained professional | 14, 22 |
| diagnostic drugs | Standardized Pollen Allergenic Extract | short ragweed pollen, sweet vernal grass pollen, orchard grass pollen, perennial rye grass pollen, timothy grass pollen, Kentucky bluegrass pollen | anaphylaxis; supervision of trained professional | 14, 22 |
| endocrine system | Androgen | danazol | embryo-fetal toxicity | 13 |
| endocrine system | Androgen | oxandrolone | hepatic injury (peliosis hepatitis; tumors); blood lipid changes and increased risk of atherosclerosis | 1, 3, 15 |
| endocrine system | Androgen | testosterone | secondary exposure to children | 13 |
| endocrine system | Androgen Receptor Inhibitor | flutamide | hepatic injury (liver failure; elevated blood values) | 1 |
| endocrine system | Androgen Receptor Inhibitor | nilutamide | interstitial pneumonitis | 5 |
| endocrine system | Cortisol Synthesis Inhibitor | levoketoconazole | hepatotoxicity; QT prolongation(ventricular dysrhythmia, torsades de pointes) | 1, 3 |
| ***Drug category*** | ***Drug class*** | ***Drug*** | ***Black box warning*** | ***Adverse reaction*** |
| endocrine system | Estrogen | estradiol | cardiovascular risk associated with smoking | 3 |
| endocrine system | Estrogen | ethinyl estradiol | cardiovascular risk associated with smoking | 3 |
| endocrine system | Estrogen | esterified | increased risk of endometrial cancer, cardiovascular events (myocardial infarction, stroke, thrombosis) and probable dementia | 3* |
| endocrine system | Estrogen | estrogens | increased risk of endometrial cancer, cardiovascular events (myocardial infarction, stroke, thrombosis) and probable dementia | 3* |
| endocrine system | Estrogen | estropipate | increased risk of endometrial cancer, cardiovascular events (myocardial infarction, stroke, thrombosis) and probable dementia | 3* |
| endocrine system | Estrogen Agonist/Antagonist | ospemifene | increased risk of endometrial cancer and cardiovascular events (myocardial infarction, stroke, thrombosis) | 3* |
| endocrine system | Estrogen Agonist/Antagonist | raloxifene | increased risk of venous thromboembolism and stroke | 3 |
| endocrine system | Estrogen Agonist/Antagonist | toremifene | QT prolongation; drug interactions (CYP3A4) | 3, 19 |
| endocrine system | Estrogen Agonist/Antagonist | tamoxifen | uterine malignancies, stroke and pulmonary embolism | 3* |
| endocrine system | Gonadotropin Releasing Hormone Receptor Antagonist | elagolix | thromboembolic disorders and vascular events | 3 |
| endocrine system | Gonadotropin Releasing Hormone Receptor Antagonist | relugolix | thromboembolic disorders and vascular events | 3 |
| endocrine system | Leptin Analog | metreleptin | risk of anti-metreleptin antibodies with neutralizing activity; lymphoma | 14, 16 |
| endocrine system | l-Thyroxine | levothyroxine | contraindicated in treatment of obesity or weight los; toxicity and adverse effects in overdose | 15 |
| endocrine system | l-Triiodothyronine | liothyronine | contraindicated in treatment of obesity or weight los; toxicity and adverse effects in overdose | 15 |
| endocrine system | Oxytocic | oxytocin | contraindicated in elective induction of labour | 13 |
| endocrine system | Parathyroid Hormone | parythyroid hormone | risk of osteosarcoma | 16 |
| endocrine system | Progesterone | progesterone | increased risk of endometrial cancer, cardiovascular events (myocardial infarction, stroke, thrombosis) and probable dementia | 3* |
| endocrine system | Progestin | norgestrel | cardiovascular risk associated with smoking | 3 |
| endocrine system | Progestin | desogestrel | cardiovascular risk associated with smoking | 3 |
| endocrine system | Progestin | dienogest | cardiovascular risk associated with smoking | 3 |
| endocrine system | Progestin | drospirenone | cardiovascular risk associated with smoking | 3 |
| endocrine system | Progestin | ethynodiol | cardiovascular risk associated with smoking | 3 |
| endocrine system | Progestin | norgestimate | cardiovascular risk associated with smoking | 3 |
| endocrine system | Progestin | norelgestromin | cardiovascular risk associated with smoking; contraindicated in obese women with BMI exceeding 30 (high risk of thromboembolic events) | 3 |
| endocrine system | Progestin | norethindrone | increased risk of endometrial cancer, cardiovascular events (myocardial infarction, stroke, thrombosis) and probable dementia | 3* |
| endocrine system | Progestin | medroxyprogesterone | loss of bone mineral density | 15 |
| ***Drug category*** | ***Drug class*** | ***Drug*** | ***Black box warning*** | ***Adverse reaction*** |
| endocrine system | Progestin Antagonist | mifepristone | termination of pregnancy | 13 |
| endocrine system | Prostaglandin E1 Analog | misoprostol | risk of uterine rupture, abortion, premature birth and birth defects; contraindicated in pregnancy | 13 |
| endocrine system | Thyroid Hormone Synthesis Inhibitor | propylthiouracil | hepatotoxicity (liver injury and acute liver failure) | 1 |
| hemostasis and blood products | Anti-inhibitor Coagulant Complex | Anti-inhibitor Coagulant Complex | embolic and thrombotic events | 3 |
| hemostasis and blood products | Antithrombin-directed RNA Interaction | fitusiran | thrombotic events; acute and recurrent gallbladder disease | 3, 8 |
| hemostasis and blood products | Blood Coagulation Factor | Prothrombin | arterial and venous thromboembolic complications in reversing VKA | 3 |
| hemostasis and blood products | Blood Coagulation Factor | coagulation Factor IX human | arterial and venous thromboembolic complications in reversing VKA | 3 |
| hemostasis and blood products | Blood Coagulation Factor | coagulation Factor X human | arterial and venous thromboembolic complications in reversing VKA | 3 |
| hemostasis and blood products | Blood Coagulation Factor | thrombin | bleeding risk or thrombosis (development of antibodies) | 3, 11 |
| hemostasis and blood products | Blood Coagulation Factor | coagulation factor VIIa recombinant human | thrombosis | 3 |
| hemostasis and blood products | Blood Coagulation Factor | emicizumab | thrombotic microangiopathy and thromboembolism | 3 |
| hemostasis and blood products | Direct Thrombin Inhibitor | droperidol | QT prolongation and torsade de pointes | 3 |
| hemostasis and blood products | Erythropoiesis-stimulating Agent | darbepoetin alfa | increased risk of mortality, myocardial infarction, stroke, venous thromboembolism and thrombosis of vascular access; tumor progression or recurrence | 16, 17 |
| hemostasis and blood products | Erythropoiesis-stimulating Agent | epoetin | increased risk of mortality, myocardial infarction, stroke, venous thromboembolism and thrombosis of vascular access; tumor progression or recurrence | 16, 17 |
| hemostasis and blood products | Erythropoiesis-stimulating Agent | erythropoetin | increased risk of mortality, myocardial infarction, stroke, venous thromboembolism and thrombosis of vascular access; tumor progression or recurrence | 16, 17 |
| hemostasis and blood products | Factor Xa Inhibitor | fondaparinux | epidural or spinal hematoma neuraxial anesthesia or spinal puncture (resulting in paralysis) | 4, 11 |
| hemostasis and blood products | Factor Xa Inhibitor | apixaban | premature discontinuation of oral anticoagulants increases risk of thrombotic events; epidural or spinal hematoma neuraxial anesthesia or spinal puncture (resulting in paralysis) | 4, 11, 20 |
| hemostasis and blood products | Factor Xa Inhibitor | rivaroxaban | premature discontinuation of oral anticoagulants increases risk of thrombotic events; epidural or spinal hematoma neuraxial anesthesia or spinal puncture (resulting in paralysis) | 4, 11, 20 |
| hemostasis and blood products | Factor Xa Inhibitor | edoxaban | premature discontinuation of oral anticoagulants increases risk of thrombotic events; epidural or spinal hematoma neuraxial anesthesia or spinal puncture (resulting in paralysis); reduced efficacy in impaired renal function | 4, 11, 20 |
| hemostasis and blood products | Heparin Reversal Agent | protamine | allergic reaction (hypotension, pulmonary vasoconstriction, pulmonary hypertension) | 14 |
| hemostasis and blood products | Hypoxia-inducible Factor Prolyl Hydroxylase Inhibitor | daprodustat | increased risk of mortality, myocardial infarction, stroke, venous thromboembolism and thrombosis of vascular access | 3, 4, 17 |
| hemostasis and blood products | Hypoxia-inducible Factor Prolyl Hydroxylase Inhibitor | vadadustat | increased risk of mortality, myocardial infarction, stroke, venous thromboembolism and thrombosis of vascular access | 3, 4, 17 |
| ***Drug category*** | ***Drug class*** | ***Drug*** | ***Black box warning*** | ***Adverse reaction*** |
| hemostasis and blood products | Iron Chelator | deferiprone | agranulocytosis and neutropenia; serious infections | 11, 21 |
| hemostasis and blood products | Iron Chelator | deferasirox | hepatotoxity; nephrotoxity (acute kidney injury, Fanconi syndrome); gastrointestinal hemorrhage | 1, 2, 8 |
| hemostasis and blood products | Iron Replacement | ferrous cation | fatal poisoning in overdose (children under 6 years) | 15 |
| hemostasis and blood products | Low Molecular Weight Heparin | enoxaparin | epidural or spinal hematoma neuraxial anesthesia or spinal puncture (resulting in paralysis) | 4, 11 |
| hemostasis and blood products | Low Molecular Weight Heparin | tinzaparin | epidural or spinal hematoma neuraxial anesthesia or spinal puncture (resulting in paralysis) | 4, 11 |
| hemostasis and blood products | Low Molecular Weight Heparin | dalteparin | spinal/epidural hematomas in neuraxial anesthesia or spinal puncture (resulting in paralysis) | 4, 11 |
| hemostasis and blood products | Lysine Analog | tranexamic acid | risk of medication errors and severe adverse effects (only intravenous use) | 22 |
| hemostasis and blood products | Natural Anticoagulant Cofaktor | Protein S | arterial and venous thromboembolic complications in reversing VKA | 3 |
| hemostasis and blood products | P2Y12 Platelet Inhibitor | prasugrel | bleeding risk | 11 |
| hemostasis and blood products | P2Y12 Platelet Inhibitor | ticagrelor | bleeding risk | 11 |
| hemostasis and blood products | P2Y12 Platelet Inhibitor | clopidogrel | diminished antiplatelet effect in patients with loss-of-function in CYP2C19 | 11 |
| hemostasis and blood products | Parenteral Iron Replacement | ferumoxytol | hypersensitivity reactions and anaphylaxis | 14 |
| hemostasis and blood products | Plasma Volume Expander | hetastarch | increased risk of mortality; acute kidney injury; coagulopathy | 2, 11, 17 |
| hemostasis and blood products | Platelet Aggregation Inhibitor | ticlopidine | hematological adverse reaction (neutropenia/agranulocytosis, TTP, aplastic anemia) | 11 |
| hemostasis and blood products | Thrombopoietin Receptor Agonist | eltrombopag | hepatotoxicity; hepatitis decompensation in patients with chronic hepatitis C | 1, 20 |
| hemostasis and blood products | Vitamin K | phytonadione | hypersensitivity reactions in intravenous and intramuscular use | 14 |
| hemostasis and blood products | Vitamin K Antagonist | warfarin | bleeding risk | 3 |
| hemostasis and blood products | Vitamin K-dependent Plasma Protein | Protein C | arterial and venous thromboembolic complications in reversing VKA | 3 |
| immunomodulators, immunotherapies and immunization | Alkalinizing Agent | brexucabtagene | cytokine release syndrome; neurologic toxicity; malignancies (particularly T cell malignancies) | 3, 4, 16 |
| immunomodulators, immunotherapies and immunization | Allogeneic Cord Blood Hematopoietic Progenitor Cell Therapy | human cord blood hematopoietic progenitor cell | fatal infusion reaction; graft-vs-host disease; engraftment syndrome; graft failure | 14 |
| immunomodulators, immunotherapies and immunization | Allogeneic Cord Blood Hematopoietic Progenitor Cell Therapy | elivaldogene | hematologic malignancy (myelodysplastic syndrome, acute myeloid leukemia) | 16 |
| ***Drug category*** | ***Drug class*** | ***Drug*** | ***Black box warning*** | ***Adverse reaction*** |
| immunomodulators, immunotherapies and immunization | Amyloid Beta-directed Antibody | lecanemab | amyloid related imaging abnormalities (edema and hemosiderin deposition; intracerebral hemorrhages; focal neurologic deficits; particularly ApoE e4 homozygotes) | 4 |
| immunomodulators, immunotherapies and immunization | Amyloid Beta-directed Antibody | donanemab | amyloid related imaging abnormalities (edema and hemosiderin deposition; intracerebral hemorrhages; focal neurologic deficits; particularly ApoE e4 homozygotes) | 4 |
| immunomodulators, immunotherapies and immunization | Anthrax Protective Antigen-directed Antibody | obiltoxaximab | hypersensitivity reactions | 14 |
| immunomodulators, immunotherapies and immunization | Anthrax Protective Antigen-directed Antibody | raxibacumab | hypersensitivity reactions | 14 |
| immunomodulators, immunotherapies and immunization | Anti-IgE | omalizumab | anaphylaxis (bronchospasm, hypotension, syncope, urticaria, angioedema) | 14 |
| immunomodulators, immunotherapies and immunization | Antimetabolite Immunosuppressant | mycophenolic acid | embryo-fetal toxicity; malignancies (particularly lymphoma and skin cancer); serious infections | 13, 16, 21 |
| immunomodulators, immunotherapies and immunization | Bispecific CD19-directed CD3-directed T Cell Engager | blinatumomab | cytokine release syndrome; neurological toxicity and immune effector cell-associated neurotoxicity syndrome | 3, 4, 11 |
| immunomodulators, immunotherapies and immunization | Bispecific CD20-directed CD3 T Cell Engager | glofitamab | cytokine release syndrome | 3 |
| immunomodulators, immunotherapies and immunization | Bispecific CD20-directed CD3 T Cell Engager | mosunetuzumab | cytokine release syndrome | 3 |
| immunomodulators, immunotherapies and immunization | Bispecific CD20-directed CD3 T Cell Engager | epcoritamab | cytokine release syndrome; neurological toxicity and immune effector cell-associated neurotoxicity syndrome | 3, 4, 11 |
| immunomodulators, immunotherapies and immunization | Bispecific CD20-directed CD3 T Cell Engager | elranatamab | cytokine release syndrome; neurological toxicity and immune effector cell-associated neurotoxicity syndrome | 3, 4, 11 |
| immunomodulators, immunotherapies and immunization | Bispecific CD20-directed CD3 T Cell Engager | linvoseltamab | cytokine release syndrome; neurological toxicity and immune effector cell-associated neurotoxicity syndrome | 3, 4, 11 |
| immunomodulators, immunotherapies and immunization | Bispecific gp100 Peptide-HLA-directed CD3 T Cell Engager | tebentafusp | cytokine release syndrome | 3 |
| immunomodulators, immunotherapies and immunization | Bispecific HER2-directed Antibody | zanidatamab | embryo-fetal toxicity | 13 |
| ***Drug category*** | ***Drug class*** | ***Drug*** | ***Black box warning*** | ***Adverse reaction*** |
| immunomodulators, immunotherapies and immunization | Bispecific HER2-directed Antibody | zenocutuzumab | embryo-fetal toxicity | 13 |
| immunomodulators, immunotherapies and immunization | Calcineurin Inhibitor Immunosuppressant | tacrolimus | malignancies; serious infections | 16, 21 |
| immunomodulators, immunotherapies and immunization | Calcineurin Inhibitor Immunosuppressant | voclosporin | malignancies; serious infections | 16, 21 |
| immunomodulators, immunotherapies and immunization | Calcineurin Inhibitor Immunosuppressant | pimecrolimus | no established long-term safety; malignancy (particularly lymphoma and skin cancer) | 16 |
| immunomodulators, immunotherapies and immunization | Calcineurin Inhibitor Immunosuppressant | cyclosporine | supervision of trained professional and availability of adequate facilities; infections; malignancies (particularly lymphoma); different preparations not bioequivalent | 16, 17, 21, 22 |
| immunomodulators, immunotherapies and immunization | CAR-T cell therapy | afamitresgene autoleucel | cytokine release syndrome | 11 |
| immunomodulators, immunotherapies and immunization | CAR-T cell therapy | brincidofovir | increased risk for mortality in long-term use | 17 |
| immunomodulators, immunotherapies and immunization | CAR-T cell therapy | lifileucel | increased treatment-related mortality; prolonged severe cytopenia; severe infection; cardiopulmonary and renal impairment; supervision by trained professional and availability of adequate facilities | 2, 3, 5, 11, 17, 21, 22 |
| immunomodulators, immunotherapies and immunization | CCR5 Co-receptor Antagonist | maraviroc | hepatotoxicity; allergic reaction | 1, 14 |
| immunomodulators, immunotherapies and immunization | CD19-directed Chimeric Antigen Receptor | ciltacabtagene | cytokine release syndrome; neurologic toxicity (ICANS); secondary hematological malignancies; hemophagocytic lymphohistiocytosis/macrophage activation syndrome; prolonged and recurrent cytopenia; immune effector cell-associated enterocolitis | 4, 11, 14, 16 |
| immunomodulators, immunotherapies and immunization | CD19-directed Chimeric Antigen Receptor | axicabtagene ciloleucel | cytokine release syndrome; neurologic toxicity; secondary hematological malignancies | 4, 14, 16 |
| immunomodulators, immunotherapies and immunization | CD19-directed Chimeric Antigen Receptor | tisagenlecleucel | cytokine release syndrome; neurologic toxicity; secondary hematological malignancies | 4, 14, 16 |
| immunomodulators, immunotherapies and immunization | CD20-directed Cytolytic Antibody | rituximab | fatal infusion-related reactions and severe mucocutaneous reactions; hepatitis B virus reactivation (hepatitis, hepatic failure); progressive multifocal leukoencephalopathy | 4, 9, 14, 20 |
| immunomodulators, immunotherapies and immunization | CD20-directed Cytolytic Antibody | obinutuzumab | hepatitis B virus reactivation (hepatitis, hepatic failure); progressive multifocal leukoencephalopathy | 4, 21 |
| ***Drug category*** | ***Drug class*** | ***Drug*** | ***Black box warning*** | ***Adverse reaction*** |
| immunomodulators, immunotherapies and immunization | CD20-directed Cytolytic Antibody | ofatumumab | hepatitis B virus reactivation (hepatitis, hepatic failure); progressive multifocal leukoencephalopathy | 4, 21 |
| immunomodulators, immunotherapies and immunization | CD22-directed Immunoconjugate | inotuzumab | hepatotoxicity and hepatic veno-occlusive disease; increased risk of post-hematopoietic stem cell transplant non-relapse mortality | 1, 17 |
| immunomodulators, immunotherapies and immunization | CD22-directed Immunoconjugate | sacituzumab govitecan | neutropenia; diarrhea | 8, 11 |
| immunomodulators, immunotherapies and immunization | CD25-directed Cytotoxin | denileukin | capillary leak syndrome | 3 |
| immunomodulators, immunotherapies and immunization | CD30-directed Immunoconjugate | brentuximab | progressive multifocal leukoencephalopathy in JC virus infection | 4, 21 |
| immunomodulators, immunotherapies and immunization | CD33-directed Immunoconjugate | gemtuzumab | hepatotoxicity and veno-occlusive disease | 1 |
| immunomodulators, immunotherapies and immunization | CD52-directed Cytolytic Antibody | alemtuzumab | infusion-related reactions; cytopenia; infections | 11, 14, 21 |
| immunomodulators, immunotherapies and immunization | Chelating Agent | penicillamine | supervision of trained professional and availability of adequate facilities | 22 |
| immunomodulators, immunotherapies and immunization | Complement Inhibitor | iptacopan | infections by encapsulated bacteria (S. pneumonia, N. meningitidis, H. influenzae b) | 21 |
| immunomodulators, immunotherapies and immunization | Complement Inhibitor | danicopan | infections by encapsulated bacteria (S. pneumonia, N. meningitidis, H. influenzae b) | 21 |
| immunomodulators, immunotherapies and immunization | Complement Inhibitor | pegcetacoplan | infections by encapsulated bacteria (S. pneumonia, N. meningitidis, H. influenzae b) | 21 |
| immunomodulators, immunotherapies and immunization | Complement Inhibitor | crovalimab | infections by meningococci | 21 |
| immunomodulators, immunotherapies and immunization | Complement Inhibitor | eculizumab | infections by meningococci | 21 |
| immunomodulators, immunotherapies and immunization | Complement Inhibitor | pozelimab | infections by meningococci | 21 |
| ***Drug category*** | ***Drug class*** | ***Drug*** | ***Black box warning*** | ***Adverse reaction*** |
| immunomodulators, immunotherapies and immunization | Complement Inhibitor | ravulizumab | infections by meningococci | 21 |
| immunomodulators, immunotherapies and immunization | Complement Inhibitor | zilucoplan | infections by meningococci | 21 |
| immunomodulators, immunotherapies and immunization | COX inhibitor | celecoxib | cardiovascular events (thrombosis, myocardial infarction, stroke); gastrointestinal adverse events (bleeding, ulceration, perforation); contraindicated in coronary artery bypass graft surgery | 3, 8 |
| immunomodulators, immunotherapies and immunization | COX inhibitor | diclofenac | cardiovascular events (thrombosis, myocardial infarction, stroke); gastrointestinal adverse events (bleeding, ulceration, perforation); contraindicated in coronary artery bypass graft surgery | 3, 8 |
| immunomodulators, immunotherapies and immunization | COX inhibitor | diflunisal | cardiovascular events (thrombosis, myocardial infarction, stroke); gastrointestinal adverse events (bleeding, ulceration, perforation); contraindicated in coronary artery bypass graft surgery | 3, 8 |
| immunomodulators, immunotherapies and immunization | COX inhibitor | etodolac | cardiovascular events (thrombosis, myocardial infarction, stroke); gastrointestinal adverse events (bleeding, ulceration, perforation); contraindicated in coronary artery bypass graft surgery | 3, 8 |
| immunomodulators, immunotherapies and immunization | COX inhibitor | fenoprofen | cardiovascular events (thrombosis, myocardial infarction, stroke); gastrointestinal adverse events (bleeding, ulceration, perforation); contraindicated in coronary artery bypass graft surgery | 3, 8 |
| immunomodulators, immunotherapies and immunization | COX inhibitor | flurbiprofen | cardiovascular events (thrombosis, myocardial infarction, stroke); gastrointestinal adverse events (bleeding, ulceration, perforation); contraindicated in coronary artery bypass graft surgery | 3, 8 |
| immunomodulators, immunotherapies and immunization | COX inhibitor | ibuprofen | cardiovascular events (thrombosis, myocardial infarction, stroke); gastrointestinal adverse events (bleeding, ulceration, perforation); contraindicated in coronary artery bypass graft surgery | 3, 8 |
| immunomodulators, immunotherapies and immunization | COX inhibitor | indomethacin | cardiovascular events (thrombosis, myocardial infarction, stroke); gastrointestinal adverse events (bleeding, ulceration, perforation); contraindicated in coronary artery bypass graft surgery | 3, 8 |
| immunomodulators, immunotherapies and immunization | COX inhibitor | ketoprofen | cardiovascular events (thrombosis, myocardial infarction, stroke); gastrointestinal adverse events (bleeding, ulceration, perforation); contraindicated in coronary artery bypass graft surgery | 3, 8 |
| immunomodulators, immunotherapies and immunization | COX inhibitor | mefenamic acid | cardiovascular events (thrombosis, myocardial infarction, stroke); gastrointestinal adverse events (bleeding, ulceration, perforation); contraindicated in coronary artery bypass graft surgery | 3, 8 |
| immunomodulators, immunotherapies and immunization | COX inhibitor | meloxicam | cardiovascular events (thrombosis, myocardial infarction, stroke); gastrointestinal adverse events (bleeding, ulceration, perforation); contraindicated in coronary artery bypass graft surgery | 3, 8 |
| immunomodulators, immunotherapies and immunization | COX inhibitor | nabumetone | cardiovascular events (thrombosis, myocardial infarction, stroke); gastrointestinal adverse events (bleeding, ulceration, perforation); contraindicated in coronary artery bypass graft surgery | 3, 8 |
| ***Drug category*** | ***Drug class*** | ***Drug*** | ***Black box warning*** | ***Adverse reaction*** |
| immunomodulators, immunotherapies and immunization | COX inhibitor | naproxen | cardiovascular events (thrombosis, myocardial infarction, stroke); gastrointestinal adverse events (bleeding, ulceration, perforation); contraindicated in coronary artery bypass graft surgery | 3, 8 |
| immunomodulators, immunotherapies and immunization | COX inhibitor | oxaprozin | cardiovascular events (thrombosis, myocardial infarction, stroke); gastrointestinal adverse events (bleeding, ulceration, perforation); contraindicated in coronary artery bypass graft surgery | 3, 8 |
| immunomodulators, immunotherapies and immunization | COX inhibitor | piroxicam | cardiovascular events (thrombosis, myocardial infarction, stroke); gastrointestinal adverse events (bleeding, ulceration, perforation); contraindicated in coronary artery bypass graft surgery | 3, 8 |
| immunomodulators, immunotherapies and immunization | COX inhibitor | sulindac | cardiovascular events (thrombosis, myocardial infarction, stroke); gastrointestinal adverse events (bleeding, ulceration, perforation); contraindicated in coronary artery bypass graft surgery | 3, 8 |
| immunomodulators, immunotherapies and immunization | COX inhibitor | tolmetin | cardiovascular events (thrombosis, myocardial infarction, stroke); gastrointestinal adverse events (bleeding, ulceration, perforation); contraindicated in coronary artery bypass graft surgery | 3, 8 |
| immunomodulators, immunotherapies and immunization | COX inhibitor | meclofenamate | cardiovascular events (thrombosis, myocardial infarction, stroke); gastrointestinal adverse events (bleeding, ulceration, perforation); contraindicated in coronary artery bypass graft surgery | 3, 8 |
| immunomodulators, immunotherapies and immunization | COX inhibitor | salsalate | cardiovascular events (thrombosis, myocardial infarction, stroke); gastrointestinal adverse events (bleeding, ulceration, perforation); contraindicated in coronary artery bypass graft surgery | 3, 8 |
| immunomodulators, immunotherapies and immunization | Disease-modifying antirheumatic drug (DMARD) | auranofin | gold toxicity: erythrocytopenia, leukopenia, granulocytopenia; proteinuria and hematuria; pruritis and rash; stomatitis; persistent diarrhea; supervision by trained professional | 8, 9, 11, 22 |
| immunomodulators, immunotherapies and immunization | Glycolipid Disialoganglioside-directed Antibody | dinutuximab | infusion-related reactions; neurotoxicity (neuropathic pain, peripheral motor neuropathy) | 4, 14 |
| immunomodulators, immunotherapies and immunization | Glycolipid Disialoganglioside-directed Antibody | naxitamab | infusion-related reactions; neurotoxicity (neuropathic pain, transverse myelitis, reversible posterior leukoencephalopathy syndrome) | 4, 14 |
| immunomodulators, immunotherapies and immunization | Immunoglobulin G | equine thymocyte immune globulin | anaphylaxis | 14 |
| immunomodulators, immunotherapies and immunization | Immunoglobulin G | pemivibart | anaphylaxis | 14 |
| immunomodulators, immunotherapies and immunization | Immunoglobulin G | vaccinia immune globulin human | interactions with glucose monitoring systems (falsely high blood glucose levels) | 19 |
| immunomodulators, immunotherapies and immunization | Immunoglobulin G | Human Immunoglobulin G | thrombosis; renal dysfunction (acute renal failure, osmotic nephrosis) | 2, 3 |
| ***Drug category*** | ***Drug class*** | ***Drug*** | ***Black box warning*** | ***Adverse reaction*** |
| immunomodulators, immunotherapies and immunization | Immunomodulatory Peptide | glatiramer acetate | anaphylaxis | 14 |
| immunomodulators, immunotherapies and immunization | Integrin Receptor Antagonist | natalizumab | progressive multifocal leukoencephlaopathy (opportunistic viral infection) | 4, 21 |
| immunomodulators, immunotherapies and immunization | Interferon alpha | ropeginterferon alfa-2b | neuropsychiatric, autoimmune, ischemic and infectious adverse effects | 3, 11, 12, 21 |
| immunomodulators, immunotherapies and immunization | Interferon alpha | peginterferon alfa-2a | neuropsychiatric, autoimmune, ischemic and infectious adverse effects | 3, 11, 12, 21 |
| immunomodulators, immunotherapies and immunization | Interleukin-17 Receptor A Antagonist | brodalumab | suicidal ideation and behavior | 12 |
| immunomodulators, immunotherapies and immunization | Interleukin-2 Receptor Blocking Antibody | basiliximab | supervision of trained professional and availability of adequate facilities | 22 |
| immunomodulators, immunotherapies and immunization | Interleukin-5 Antagonist | reslizumab | anaphylaxis | 14 |
| immunomodulators, immunotherapies and immunization | Interleukin-6 Receptor Antagonist | sarilumab | serious infections (bacterial, viral, invasive fungi, opportunistic pathogens) | 21 |
| immunomodulators, immunotherapies and immunization | Interleukin-6 Receptor Antagonist | tocilizumab | serious infections (tuberculosis, bacterial, viral, invasive fungi, opportunistic pathogens) | 21 |
| immunomodulators, immunotherapies and immunization | Live Attenuated Intravesical Bacillus Calmette-Guerin Vaccine | Bacillus Calmette-guerin | risk of transmission and BCG infections; serious infections when administered intravesical | 21 |
| immunomodulators, immunotherapies and immunization | Lymphocyte Growth Factor | aldesleukin | serious infections (sepsis, bacterial endocarditis); capillary leak syndrome; neurologic toxicity (coma, permanent neurological deficits) | 3, 4, 21 |
| immunomodulators, immunotherapies and immunization | mTOR Inhibitor Immunosuppressant | everolimus | serious infections; malignancies; kidney graft thrombosis; nephrotoxicity; increased mortality in heart transplantation; supervision of trained professional | 2, 3, 14, 16, 17, 21, 22 |
| immunomodulators, immunotherapies and immunization | mTOR Inhibitor Immunosuppressant | sirolimus | serious infections; malignancies; not recommended in liver and lung transplant (increased mortality, graft loss, hepatic artery thrombosis, bronchial anastomotic dehiscence) | 16, 17, 21 |
| immunomodulators, immunotherapies and immunization | Nitroimidazole | azathioprine | malignancy (post-transplant lymphoma, hepatosplenic T-cell lymphoma) | 16 |
| ***Drug category*** | ***Drug class*** | ***Drug*** | ***Black box warning*** | ***Adverse reaction*** |
| immunomodulators, immunotherapies and immunization | Nonsteroidal Anti-inflammatory Drug | ketorolac | cardiovascular events (thrombosis, myocardial infarction, stroke); gastrointestinal adverse events (bleeding, ulceration, perforation); contraindicated in coronary artery bypass graft surgery; renal risk (contraindicated in advanced renal impairment); bleeding risk; contraindicated as prophylactic analgesic before major surgery, during labor and in combination with other COX inhibitors; dosage adjustmentt for special populations | 2, 3, 8 |
| immunomodulators, immunotherapies and immunization | Plasma Kallikrein Inhibitor | ecallantide | anaphylaxis | 14 |
| immunomodulators, immunotherapies and immunization | Pyrimidine Synthesis Inhibitor | leflunomide | hepatotoxicit; embryo-fetal toxicity | 1, 13 |
| immunomodulators, immunotherapies and immunization | Retinoid | bexarotene | embryo-fetal toxicity | 13 |
| immunomodulators, immunotherapies and immunization | Retinoid | isotretinoin | embryo-fetal toxicity | 13 |
| immunomodulators, immunotherapies and immunization | Retinoid | tretinoin | embryo-fetal toxicity; differentiation syndrome | 13, 14 |
| immunomodulators, immunotherapies and immunization | Retinoid | palovarotene | embryo-fetal toxicity; premature epiphyseal closure in growing pediatric patients | 13, 15 |
| immunomodulators, immunotherapies and immunization | Retinoid | acitretin | embryo-fetal toxicity; teratogenic | 13 |
| immunomodulators, immunotherapies and immunization | Selective T Cell Costimulation Blocker | belatacept | serious infections; malignancies; not recommended in liver transplant (graft loss, increased mortaliity); post-transplant lymphoproliferative disorder; supervision of trained professional | 11, 16, 17, 21, 22 |
| immunomodulators, immunotherapies and immunization | Thalidomide Analog | lenalidomide | embryo-fetal toxicity; hematologic toxicity (neutropenia, thromobcytopenia); thromboembolism (deep vein thrombosis, pulmonary embolism, myocardial infarction, stroke) | 3, 4, 11, 13 |
| immunomodulators, immunotherapies and immunization | Thalidomide Analog | thalidomide | embryo-fetal toxicity; thromboembolism (deep vein thrombosis, pulmonary embolism) | 3, 13 |
| immunomodulators, immunotherapies and immunization | Thalidomide Analog | pomalidomide | embryo-fetal toxicity; thromboembolism (deep vein thrombosis, pulmonary embolism, myocardial infarction, stroke) | 3, 4, 11, 13 |
| immunomodulators, immunotherapies and immunization | Tumor Necrosis Factor Inhibitor | adalimumab | serious infections (tuberculosis, bacterial sepsis, invasive fungal infections, opportunistic pathogens); malignancy (particularly lymphoma) | 16, 21 |
| ***Drug category*** | ***Drug class*** | ***Drug*** | ***Black box warning*** | ***Adverse reaction*** |
| immunomodulators, immunotherapies and immunization | Tumor Necrosis Factor Inhibitor | certolizumab | serious infections (tuberculosis, bacterial sepsis, invasive fungal infections, opportunistic pathogens); malignancy (particularly lymphoma) | 16, 21 |
| immunomodulators, immunotherapies and immunization | Tumor Necrosis Factor Inhibitor | etanercept | serious infections (tuberculosis, bacterial sepsis, invasive fungal infections, opportunistic pathogens); malignancy (particularly lymphoma) | 16, 21 |
| immunomodulators, immunotherapies and immunization | Tumor Necrosis Factor Inhibitor | golimumab | serious infections (tuberculosis, bacterial sepsis, invasive fungal infections, opportunistic pathogens); malignancy (particularly lymphoma) | 16, 21 |
| immunomodulators, immunotherapies and immunization | Tumor Necrosis Factor Inhibitor | infliximab | serious infections (tuberculosis, bacterial sepsis, invasive fungal infections, opportunistic pathogens); malignancy (particularly lymphoma) | 16, 21 |
| metabolism, gastrointestinal and respiratory system | Aldehyde Dehydrogenase Inhibitor | disulfiram | contraindicated in state of alcohol intoxication or without patient knowledge | 15, 22 |
| metabolism, gastrointestinal and respiratory system | Amylin Analog | pramlintide | severe hypoglycemia | 15 |
| metabolism, gastrointestinal and respiratory system | Biguanide | metformin | lactic acidosis | 15 |
| metabolism, gastrointestinal and respiratory system | Bile Acid | obeticholic acid | hepatic decompensation and failure in patients with primary biliary cholangitis patients with cirrhosis | 1 |
| metabolism, gastrointestinal and respiratory system | Bile Acid | chenodiol | hepatotoxicity; poor response rate in some patient subgroups; increased rate of cholecystectomy | 1, 8 |
| metabolism, gastrointestinal and respiratory system | Copper-containing Intrauterine Device | copper | risk of complications due to improper insertion | 22 |
| metabolism, gastrointestinal and respiratory system | Cystic Fibrosis Transmembrane Conductance Regulator Potentiator | deutivacaftor | hepatotoxicity (liver injury and failure) | 1 |
| metabolism, gastrointestinal and respiratory system | Cystic Fibrosis Transmembrane Conductance Regulator Potentiator | elexacaftor | hepatotoxicity (liver injury and failure) | 1 |
| metabolism, gastrointestinal and respiratory system | Cystic Fibrosis Transmembrane Conductance Regulator Potentiator | ivacaftor | hepatotoxicity (liver injury and failure) | 1 |
| metabolism, gastrointestinal and respiratory system | Cystic Fibrosis Transmembrane Conductance Regulator Potentiator | tezacaftor | hepatotoxicity (liver injury and failure) | 1 |
| ***Drug category*** | ***Drug class*** | ***Drug*** | ***Black box warning*** | ***Adverse reaction*** |
| metabolism, gastrointestinal and respiratory system | Cystic Fibrosis Transmembrane Conductance Regulator Potentiator | vanzacaftor | hepatotoxicity (liver injury and failure) | 1 |
| metabolism, gastrointestinal and respiratory system | GLP-1 Receptor Agonist | dulaglutide | risk of thyroid c-cell tumors (animal testings, uncertain for humans) | 16 |
| metabolism, gastrointestinal and respiratory system | GLP-1 Receptor Agonist | liraglutide | risk of thyroid c-cell tumors (animal testings, uncertain for humans) | 16 |
| metabolism, gastrointestinal and respiratory system | GLP-1 Receptor Agonist | semaglutide | risk of thyroid c-cell tumors (animal testings, uncertain for humans) | 16 |
| metabolism, gastrointestinal and respiratory system | GLP-1 Receptor Agonist | exenatide | risk of thyroid c-cell tumors (animal testings, uncertain for humans) | 16 |
| metabolism, gastrointestinal and respiratory system | GLP-1 Receptor Agonist | tirzepatide | risk of thyroid c-cell tumors (animal testings, uncertain for humans) | 16 |
| metabolism, gastrointestinal and respiratory system | Guanylate Cyclase-C Agonist | linaclotide | risk of serious dehydration in children (contraindicated in age below 2 years) | 15 |
| metabolism, gastrointestinal and respiratory system | Guanylate Cyclase-C Agonist | plecanatide | risk of serious dehydration in children (contraindicated in age below 2 years) | 15 |
| metabolism, gastrointestinal and respiratory system | HMG-CoA Reductase Inhibitor | simvastatin | skeletal muscle effects (myopathy, rhandomyolysis); drug interactions (CYP3A4) | 10, 19 |
| metabolism, gastrointestinal and respiratory system | Hydrolytic Lysosomal Glucocerebroside-specific Enzyme | idursulfase | anaphylaxis (respiratory distress, hypoxia, hypotension, urticaria and angioedema) | 14 |
| metabolism, gastrointestinal and respiratory system | Hydrolytic Lysosomal Glucocerebroside-specific Enzyme | galsulfase | hypersensitivity reactions and anaphylaxis | 14 |
| metabolism, gastrointestinal and respiratory system | Hydrolytic Lysosomal Glucocerebroside-specific Enzyme | imiglucerase | hypersensitivity reactions and anaphylaxis | 14 |
| metabolism, gastrointestinal and respiratory system | Hydrolytic Lysosomal Glucocerebroside-specific Enzyme | taliglucerase alfa | hypersensitivity reactions and anaphylaxis | 14 |
| metabolism, gastrointestinal and respiratory system | Hydrolytic Lysosomal Glucocerebroside-specific Enzyme | velaglucerase alfa | hypersensitivity reactions and anaphylaxis | 14 |
| ***Drug category*** | ***Drug class*** | ***Drug*** | ***Black box warning*** | ***Adverse reaction*** |
| metabolism, gastrointestinal and respiratory system | Hydrolytic Lysosomal Glucocerebroside-specific Enzyme | elosulfase alfa | hypersensitivity reactions and anaphylaxis; acute respiratory complications | 3, 5, 14 |
| metabolism, gastrointestinal and respiratory system | Hydrolytic Lysosomal Glucocerebroside-specific Enzyme | laronidase | hypersensitivity reactions and anaphylaxis; acute respiratory complications | 3, 5, 14 |
| metabolism, gastrointestinal and respiratory system | Hydrolytic Lysosomal Glucocerebroside-specific Enzyme | alglucosidase alfa | hypersensitivity reactions and anaphylaxis; immune-mediated reactions (proteinuria, nephrotic syndrome, necrotizing skin lesions); risk of acute cardiorespiratory failure | 2, 3, 5, 9, 11, 14 |
| metabolism, gastrointestinal and respiratory system | Hydrolytic Lysosomal Glucocerebroside-specific Enzyme | avalglucosidase alfa | hypersensitivity reactions and anaphylaxis; infusion-associated reactions; risk of acute cardiorespiratory failure | 3, 5, 14 |
| metabolism, gastrointestinal and respiratory system | Hydrolytic Lysosomal Neutral Glycosphingolipid-specific Enzyme | agalsidase beta | hypersensitivity reactions and anaphylaxis | 14 |
| metabolism, gastrointestinal and respiratory system | Hydrolytic Lysosomal Neutral Glycosphingolipid-specific Enzyme | pegunigalsidase alfa | hypersensitivity reactions and anaphylaxis | 14 |
| metabolism, gastrointestinal and respiratory system | Hydrolytic Lysosomal Neutral Glycosphingolipid-specific Enzyme | olipudase alfa | hypersensitivity reactions and anaphylaxis | 14 |
| metabolism, gastrointestinal and respiratory system | Hydrolytic Lysosomal Neutral Glycosphingolipid-specific Enzyme | cipaglucosidase | hypersensitivity reactions and anaphylaxis; infusion-associated reactions; risk of acute cardiorespiratory failure | 3, 5, 14 |
| metabolism, gastrointestinal and respiratory system | Hydrolytic Lysosomal N-terminal Tripeptidyl Peptidase | cerliponase alfa | hypersensitivity reactions and anaphylaxis | 14 |
| metabolism, gastrointestinal and respiratory system | Hydrolytic Lysosomal Triacylglycerol-specific Enzyme | sebelipase alfa | hypersensitivity reactions and anaphylaxis | 14 |
| metabolism, gastrointestinal and respiratory system | Insulin | insulin human | acute bronchospasm in chronic lung disease | 5 |
| metabolism, gastrointestinal and respiratory system | Leukotriene Receptor Antagonist | montelukast | neuropsychiatric events (agitation, aggression, depression, sleep disturbances, suicidality) | 12 |
| metabolism, gastrointestinal and respiratory system | Microsomal Triglyceride Transfer Protein Inhibitor | lomitapide | hepatotoxicity (elevations in transaminases; hepatic steatosis) | 1 |
| metabolism, gastrointestinal and respiratory system | Omega-3 Fatty Acid | Omega-3 Fatty Acid | potential antithrombotic effects in excessive intake (increased bleeding time and INR) | 11 |
| ***Drug category*** | ***Drug class*** | ***Drug*** | ***Black box warning*** | ***Adverse reaction*** |
| metabolism, gastrointestinal and respiratory system | Oxidation-Reduction Agent | methylene blue | serotonin syndrome in combination with serotonergic drugs and MOR agonists; drug interactions | 12, 19 |
| metabolism, gastrointestinal and respiratory system | Peroxisome Proliferator Receptor alpha Agonist | glimepiride | exacerbation of congestive heart failure (contraindicated in NYHA class III and IV) | 20 |
| metabolism, gastrointestinal and respiratory system | Peroxisome Proliferator Receptor alpha Agonist | pioglitazone | exacerbation of congestive heart failure (contraindicated in NYHA class III and IV) | 20 |
| metabolism, gastrointestinal and respiratory system | Peroxisome Proliferator Receptor alpha Agonist | rosiglitazone | exacerbation of congestive heart failure (contraindicated in NYHA class III and IV); increased risk of myocardial ischemic events (angina, myocardial infarction) | 3, 20 |
| metabolism, gastrointestinal and respiratory system | Peroxisome Proliferator Receptor alpha Agonist | gemfibrozil | increased risk of mortality (fibrates, particularly clofibrate and pharmacologically and chemically similar) | 17 |
| metabolism, gastrointestinal and respiratory system | Phenylalanine Metabolizing Enzyme | pegvaliase | anaphylaxis | 14 |
| metabolism, gastrointestinal and respiratory system | RANK Ligand Inhibitor | romosozumab | risk of myocardial infarction, stroke and cardiovascular death | 3, 4, 17 |
| metabolism, gastrointestinal and respiratory system | RANK Ligand Inhibitor | denosumab | severe hypocalcemia in advanced kidney disease | 15 |
| metabolism, gastrointestinal and respiratory system | Recombinant Human Lysosomal alpha-Mannosidase | velmanase alfa-tycv | hypersensitivity reactions and anaphylaxis | 14 |
| metabolism, gastrointestinal and respiratory system | SGLT-2-inhibitor | bexagliflozin | ketoacidosis (euglycemic, diabetic) | 15 |
| metabolism, gastrointestinal and respiratory system | SGLT-2-inhibitor | velagliflozin | ketoacidosis (euglycemic, diabetic) | 15 |
| metabolism, gastrointestinal and respiratory system | Tissue-nonspecific Alkaline Phosphatase | asfotase alfa | hypersensitivity reactions and anaphylaxis | 14 |
| metabolism, gastrointestinal and respiratory system | Uric Acid-specific Enzyme | pegloticase | hypersensitivity reactions and anaphylaxis; G6PD deficiency associated hemolysis and methemoglobinemia (contraindicated in G6PD deficiency) | 11, 14 |
| metabolism, gastrointestinal and respiratory system | Xanthine Oxidase Inhibitor | febuxostat | increased risk of cardiovascular death (in established cardiovascular disease) | 17 |
